# Glucocorticoid treatment influences prostate cancer cell growth and the tumor microenvironment via altered glucocorticoid receptor signaling in prostate fibroblasts

**DOI:** 10.1038/s41388-023-02901-5

**Published:** 2023-11-29

**Authors:** Andrea Eigentler, Florian Handle, Silvia Schanung, Antonia Degen, Hubert Hackl, Holger H. H. Erb, Georgios Fotakis, Julia Hoefer, Christian Ploner, Karin Jöhrer, Isabel Heidegger, Andreas Pircher, Werner Klotz, Manfred Herold, Georg Schäfer, Zoran Culig, Martin Puhr

**Affiliations:** 1 Department of Urology, Medical University of Innsbruck, Innsbruck, Austria; 2 Institute of Pathology, Neuropathology and Molecular Pathology, Medical University of Innsbruck, Innsbruck, Austria; 3 Institute of Bioinformatics, Biocenter, Medical University of Innsbruck, Innsbruck, Austria; 4 Department of Urology, Faculty of Medicine, University Hospital Carl Gustav Carus, Technische Universität Dresden, Dresden, Germany; 5 Department of Plastic, Reconstructive and Aesthetic Surgery, Medical University of Innsbruck, Innsbruck, Austria; 6 Innovacell GesmbH, Mitterweg 25, Innsbruck, Austria; 7 Department of Internal Medicine V, Medical University of Innsbruck, Innsbruck, Austria; 8 Department of Internal Medicine II, Medical University of Innsbruck, Innsbruck, Austria

**Keywords:** Prostate cancer, Hormone receptors, Extracellular matrix, Extracellular signalling molecules

## Abstract

Despite significant therapeutic advances in recent years, treatment of metastatic prostate cancer (PCa) remains palliative, owing to the inevitable occurrence of drug resistance. There is increasing evidence that epithelial glucocorticoid receptor (GR) signaling and changes in the tumor-microenvironment (TME) play important roles in this process. Since glucocorticoids (GCs) are used as concomitant medications in the course of PCa treatment, it is essential to investigate the impact of GCs on stromal GR signaling in the TME. Therefore, general GR mRNA and protein expression was assessed in radical prostatectomy specimens and metastatic lesions. Elevated stromal GR signaling after GC treatment resulted in altered GR-target gene, soluble protein expression, and in a morphology change of immortalized and primary isolated cancer-associated fibroblasts (CAFs). Subsequently, these changes affected proliferation, colony formation, and 3D-spheroid growth of multiple epithelial PCa cell models. Altered expression of extra-cellular matrix (ECM) and adhesion-related proteins led to an ECM remodeling. Notably, androgen receptor pathway inhibitor treatments did not affect CAF viability. Our findings demonstrate that GC-mediated elevated GR signaling has a major impact on the CAF secretome and the ECM architecture. GC-treated fibroblasts significantly influence epithelial tumor cell growth and must be considered in future therapeutic strategies.

## Introduction

Drug resistance is associated with accelerated tumor progression and remains a significant challenge in the treatment of metastatic prostate cancer (PCa). Despite the recent introduction of novel hormone therapies (NHT), taxane-based chemotherapy, PARP inhibitors, or radio-ligand therapies, metastatic PCa treatment remains palliative, claiming the urgent need to identify the underlying molecular mechanisms for therapy insensitivities. Although the androgen receptor (AR) is the primary therapeutic target for PCa, and changes in AR activity are associated with the emergence of therapy resistance, the underlying biological mechanisms remain elusive [1, 2]. In addition to AR, we and others have identified increased epithelial glucocorticoid receptor (GR) expression, glucocorticoid (GC) medication, and consequently increased GR signaling with NHT and chemotherapy resistance during the last years [3–5]. Based on these findings, altered epithelial GR signaling and the mutually activated AR-GR transcriptome network have been identified as crucial survival mechanisms for epithelial PCa cells, bypassing NHT [6–8].

Although these findings shed new light on this topic, past and current research has mainly focused on the effects of systemic GC application in PCa patients, epithelial GR signaling, and its impact on tumor progression. However, little is known about the direct GC-mediated effects on GR activity in the tumor-microenvironment (TME), especially in cancer-associated fibroblasts (CAFs), which are known to be even involved in tumor progression [9–11]. Possible functional consequences on the stromal compartment and paracrine effects on the prostate epithelium may influence tumor growth. GCs may be an additional yet unknown reason for the development of therapy resistance. In this context, we recently identified monoamine oxidase A (MAO-A) as a mutually directly upregulated druggable epithelial and stromal GR target [7]. Additionally, MAO-A is a major driver of PCa cell growth and resistance to therapy [7]. Since GCs are routinely administered systemically as concomitant medications in the course of abiraterone, docetaxel, and cabazitaxel treatment [12–14], we hypothesized that GCs may also influence the stromal GR axis in the TME. Therefore, the present study specifically addressed the impact of GC treatment and elevated GR activity in immortalized and primary isolated fibroblasts. In summary, we report a significantly altered fibroblast secretome, as well as an adhesion and extracellular matrix (ECM) expression profile after GC treatment, with direct consequences for accelerated PCa cell growth.

## Results

### Single-cell mapping and IHC identify a prominent stromal GR signature in benign and PCa tissue

**Fig. 1:**
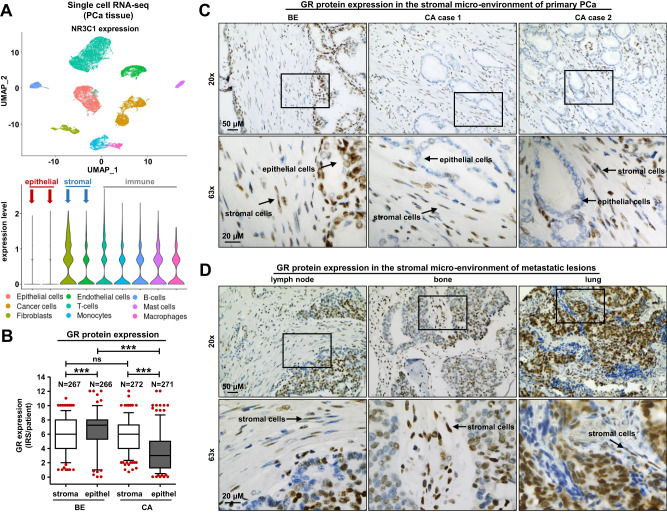
GR mRNA and protein expression in benign and malign prostate tissue. **A** Single-cell RNA-seq from 4 PCa patients and t-SNE analysis of 24.625 cells freshly isolated PCa cells, as well as a violin blot for NR3C1 expression levels in identified and selected cell subpopulations. **B** Quantification of stromal and epithelial GR immune-reactivity scores (IRS) in benign and malign tissue specimens, including 280 PCa patients after IHC staining for GR (one-way ANOVA and correction for multiple testing using Dunn’s comparison test; ***P < 0.001; Box Whisker Plot with 5–95 percentile). **C**, **D** Representative microscopy images of benign and primary PCa tissues and diverse metastatic lesions for stromal and epithelial GR expression. Magnification: ×20 (scale bar = 50 µm) and ×63 (scale bar = 20 µm).

To investigate the heterogeneity of GR (NR3C1) mRNA expression across the various cell types found in the prostate, we re-analyzed our GSE193337 scRNA-seq data [15] as well as publicly available scRNA-seq datasets representing benign and BPH prostates [16]. Dimension reduction, clustering, and annotation of the dataset containing 24.625 cells from four PCa patients revealed a clear separation of the cell types (Fig. 1A). GR expression varied strongly, with very low mRNA expression in epithelial cells, in contrast to intermediate or strong expression in the TME represented by fibroblasts, endothelial cells, and immune cells (T cells, monocytes, B cells, mast cells, and macrophages) (Fig. 1A). Compared to the PCa dataset, public scRNA-seq data from normal and benign prostate hyperplasia (BPH) prostate glands identified more prominent GR mRNA levels in epithelial cells, fibroblasts, smooth muscle, endothelial, and immune cells. Conversely, basal epithelial, hillock, club, and neuroendocrine cells showed weaker expression (Fig. S1A). Further experiments successfully translated the mRNA observations to the protein level. Briefly, IHC and quantitative analysis of 280 treatment-naïve PCa patients showed an intermediate or strong and constant GR staining in the stromal compartment in both benign and malignant tissue sections, in contrast to a significantly reduced epithelial GR staining in primary PCa compared to benign prostate glands (Fig. 1B, C). Furthermore, IHC screening of different PCa metastatic lesions (lymph node, bone, and lung) revealed unchanged strong GR staining in the TME, concluding that stromal GR expression is consistently prominent in the benign prostate and does not significantly alter during PCa progression compared to epithelial GR expression (Fig. 1D).

### GR is not essential for prostate fibroblast survival but is functionally active

**Fig. 2:**
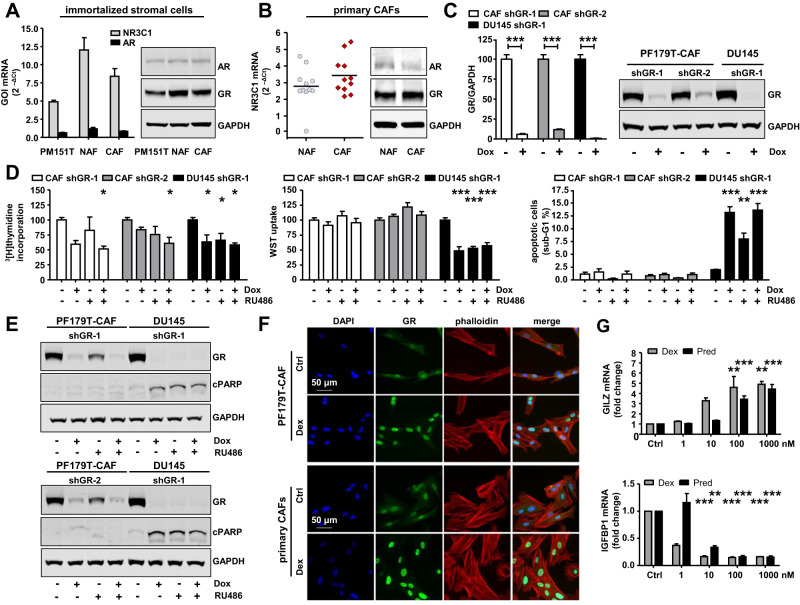
GR is not essential for prostate fibroblast survival but is functionally active. **A**, **B** AR/GR mRNA and protein expression in benign and malign immortalized stromal prostate cell lines and in primary isolated and cultured NAFs and CAFs. **C** Representative Western blot bands, as well as quantification of reduced GR expression after 6 d transient GR knockdown with specific doxycycline-inducible shRNA sequences shGR-1 and shGR-2 in PF179T-CAF-shGR-1/shGR-2 and DU145-shGR-1 cells. Data represent mean + SEM from at least 3 independent experiments (unpaired *t*-test; ***P < 0.001). **D** Measurement of altered cell proliferation, cell viability, as well as apoptosis with ^3^[H]-thymidine incorporation, WST uptake, and PI-staining after 6 d GR single knockdown, pharmacological inhibition with RU486 or combination treatment of PF179T-CAF-shGR-1/shGR-2 and DU145-shGR-1 cells. Data represent mean + SEM from at least 3 independent experiments (one-way ANOVA and correction for multiple testing using Dunnett’s comparison test; *P < 0.05; ***P < 0.001). **E** Representative Western blot bands of altered GR and cPARP expression after single GR knockdown, pharmacological inhibition with RU486, or combination treatment of PF179T-CAF-shGR-1/shGR-2 and DU145-shGR-1 cells. **F** Representative immunofluorescence microscopy images of PF179T-CAF and primary isolated CAFs for cytoplasmatic and nuclear GR expression after 60 min 100 nM Dex treatment. Magnification: 40× (scale bar = 50 µm). **G** Dose-dependent altered GILZ and IGFBP1 mRNA expression after treatment with increasing concentrations of Dex and Pred (0, 1 nM, 10 nM, 100 nM, 1000 nM) for 1 d. Data represent mean + SEM from 3 independent experiments (one-way ANOVA and correction for multiple testing using Dunnett’s comparison test; **P < 0.01; ***P < 0.001).

Based on our observations in PCa tissues, we performed an in vitro screen for GR/AR mRNA and protein expression to evaluate the potential therapeutic effects of stromal GR targeting. Immortalized smooth muscle and fibroblast cell lines (PM151T, PF179T-NAF, PF179T-CAF), as well as primary isolated normal and cancer-associated fibroblasts (NAFs, CAFs), showed strong GR and weak AR expression patterns (Fig. 2A, B). Notably, single GR knockdown using the generated PF179T-CAF-shGR-1 and PF179T-CAF-shGR-2 cell sub-lines, as well as pharmacological GR inhibition with RU486, did not result in a constant reliably reduced cell proliferation, growth, viability, or elevated apoptosis in both models, compared to DU145-shGR-1 cells, representing the epithelial compartment (Fig. 2C, D, Fig. S1B, C). The authors want to point out that RU486 is not a specific GR inhibitor. RU486 can also inhibit the progesterone receptor (PR). However, it was already shown in a previous publication that the PR is neither expressed at mRNA nor at protein level in used fibroblast cultures [4]. Only a combination of GR knockdown and pharmacological inhibition resulted in reduced cell proliferation, as measured by ^3^[H]-thymidine incorporation, but not in altered cell viability nor apoptosis, as confirmed by the unchanged cPARP expression in either fibroblast cell line (Fig. 2E, Fig. S1 D). The number of cells in S-phase (^3^[H]-thymidine incorporation) was reduced by nearly 50% upon shGR-1 induction by Dox treatment in presence and absence of RU486 in PF179T-CAF and DU145 cells. However, GR knockdown alone failed to reach our significance threshold, and knockdown with a second shRNA (shGR-2) yielded much weaker results. In addition, RU486 mono-treatment showed weaker effects in PF179T-CAF than in DU145. Measurement of metabolic potential (WST uptake) and apoptosis (sub-G1) revealed that DU145 cells were significantly affected by GR knockdown and biochemical inhibition, whereas PF179T-CAF cells did not show any effect. Next, we aimed to clarify whether GR is active in these cells. Short-term GC treatment (1 h) resulted in GR activation and translocation to the nucleus in both immortalized and primary CAFs (Fig. 2F, Fig. S2A). Furthermore, treatment with increasing concentrations of dexamethasone (Dex) and prednisolone (Pred) significantly altered GILZ and IGFBP1 mRNA expression, which are known as positively and negatively regulated GR-target genes. Therefore, we conclude that GR is highly expressed and functionally active in the investigated fibroblasts (Fig. 2G).

### Identification of the stromal GR signature

**Fig. 3:**
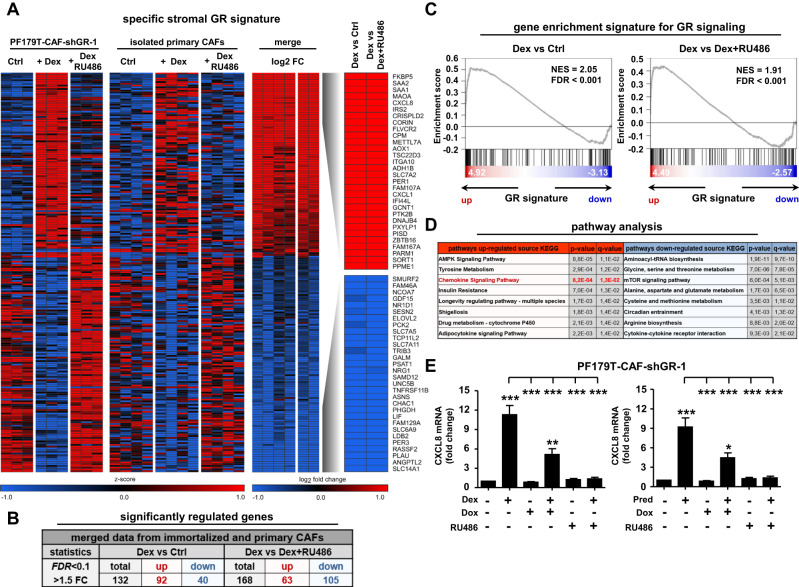
Identification of the stromal GR-target gene signature after GC treatment. **A** Heat map of top 30 mutual significantly upregulated and downregulated GR-target genes using immortalized PF179T-CAF-shGR-1 cells and primary isolated CAFs from 4 PCa patients. **B** General significantly upregulated and downregulated GR-target genes (FDR < 0.1, > 1.5 FC), after control DMSO treatment, as well as after single 100 nM Dex, or combined 100 nM Dex and 12 µM RU486 treatment for 1 d, using PF179T-CAF-shGR-1 cells and primary isolated CAFs. **C** Gene-set enrichment blots for significantly elevated GR signatures comparing Dex versus control (NES = 2.05, FDR < 0.001), or Dex versus Dex + RU486 (NES = 1.91, FDR < 0.001) treatments using stromal PF179T-CAF-shGR-1 and primary isolated CAF datasets. **D** KEGG and WIKI pathway analysis for the identification of significantly regulated pathways after GC treatment. **E** Representative confirmation of Affymetrix array results by measurement of significantly altered CXCL8 mRNA expression in PF179T-CAF-shGR-1 cells using qRT-PCR after single 100 nM Dex, 100 nM Pred, 1 µg/ml Dox, 12 µM RU486, or combination treatments for 1 d. Data represent mean + SEM from 3 independent experiments (one-way ANOVA and correction for multiple testing using Bonferroni’s comparison test; *P < 0.05; **P < 0.01; ***P < 0.001).

Since GR was functionally active in the investigated fibroblasts, we next assessed the altered stromal GR signature after GC treatment. Re-analysis of microarray data from PF179T-CAF-shGR-1 cells [7], as well as including primary CAFs from four PCa patients after GR activation and combined activation/pharmacological inhibition, revealed 132 specifically regulated (92 upregulated and 40 downregulated; Dex versus Ctrl) and 168 specifically regulated (63 upregulated and 105 downregulated; Dex versus Dex + RU486) GR-target genes after 24 h of GC treatment (Fig. 3A, B). In line with these findings, all datasets upon Dex treatment displayed enrichment of the known GR signature (Fig. 3C). Interestingly, GR-target gene identification, followed by subsequent pathway analysis revealed significantly elevated chemokine signaling [kyoto encyclopedia of genes and genomes (KEGG) pathway analysis] after Dex treatment (Fig. 3D). The GR-dependent elevated chemokine signaling signature after GC treatment was confirmed by assessing altered IL-8 (CXCL8) mRNA expression after induction with Dex or Pred alone, after combined induction and pharmacological inhibition, and combined induction and GR knockdown using PF179T-CAF-shGR-1 cells (Fig. 3E). In this context, screening of primary isolated CAFs after induction with Dex or Pred alone or in combination with pharmacological GR inhibition showed similar results (Fig. S2B).

### Altered chemokine signaling results in accelerated cancer cell growth

**Fig. 4:**
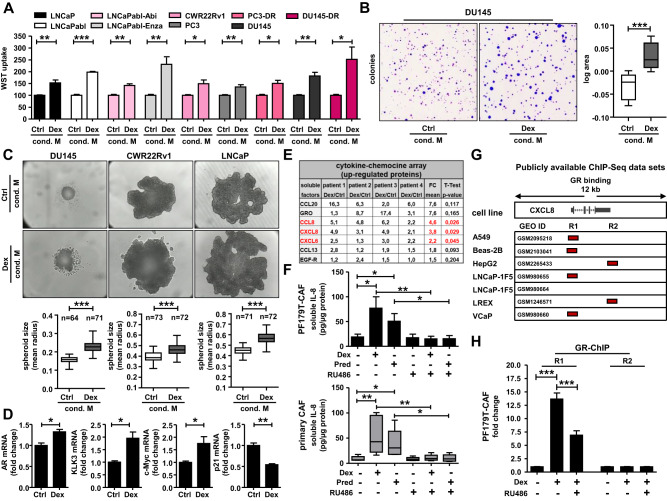
Altered chemokine signaling results in accelerated cancer cell growth. **A** Measurement of elevated cell proliferation/viability after 4 d incubation of several epithelial PCa cell lines, representing different tumor stages with collected conditioned stromal medium of PF179T-CAFs after 3 d control or 100 nM Dex treatment. Data represent mean + SEM from 3 independent experiments (unpaired *t*-test; *P < 0.05; **P < 0.01; ***P < 0.001). **B** Significantly increased DU145 colonies size after 12 d culture with collected conditioned stromal PF179T-CAF medium after previously 3 d control or 100 nM Dex treatment. Data represent the mean log area of 5616 measured colonies from 5 independent experiments (paired *t*-test; ***P < 0.001; Box Whisker Plot with min-max percentile). **C** Elevated 3D-spheroid growth of DU145, CWR22Rv1, and LNCaP cells after 8 d culture with collected conditioned stromal PF179T-CAF medium after previously 3 d control or 100 nM Dex treatment. Data represent mean area measured from 3 independent experiments (paired *t*-test; *P < 0.05; **P < 0.01; ***P < 0.001, Box Whisker Plot with min–max percentile). **D** Observed elevated AR, KLK3, and c-Myc, as well as decreased p21 mRNA expression after 8 d LNCaP spheroid growth with collected conditioned stromal PF179T-CAF medium after previously 3 d control or 100 nM Dex treatment. Data represent mean + SEM from 3 independent experiments (unpaired *t*-test; *P < 0.05; **P < 0.01). **E** Measurement of elevated soluble factors using commercially available cytokine and chemokine protein arrays after 3 d 100 nM Dex treatment using primary isolated CAFs from 4 PCa patients. Data represent mean fold change (paired t-test; marked in red, P < 0.05). **F** Altered IL-8 protein expression in PF179T-CAF cells and primary isolated CAFs after 3 d single/combination treatments with 100 nM Dex, 100 nM Pred, and 6 µM RU486, using specific IL-8-coupled magnetic Luminex^®^ beads. Data represent mean + SEM from 3 independent experiments (one-way ANOVA and correction for multiple testing using Bonferroni’s comparison test; *P < 0.05; **P < 0.01). **G** Identification of 2 GR-binding sites (R1 and R2) near the CXCL8 gene in A549, Beas-2B, HepG2, LNCaP-1F5, LREX, and VCaP, cells, screening publicly available ChIP-Seq datasets. **H** GR-ChIP using PF179T-CAF cells after treatment with 100 nM Dex alone or with 6 µM RU486 for 16 h. Data represent mean + SEM from 3 independent experiments (one-way ANOVA and correction for multiple testing using Bonferroni’s comparison test; ***P < 0.001).

To test the potential functional effect of altered stromal chemokine signaling on the epithelial compartment after GC treatment, we collected the supernatant of PF179T-CAF cells after 3 d incubation with Dex. Moreover, we cultured several PCa cell models representing different disease stages in this medium for 4 d (Fig. S2C). WST uptake revealed significantly elevated cell proliferation/viability in LNCaP, androgen-ablated LNCaPabl, abiraterone- and enzalutamide-resistant LNCaPabl-Abi/Enza, CWR22Rv1, PC3, DU145, docetaxel-resistant PC3-DR, and DU145-DR cells (Fig. 4A). A possible non-specific influence of Dex residues in the medium could be excluded in a control proliferation experiment, showing that treatment of LNCaP cells with increasing Dex concentrations had no significant impact on LNCaP cell proliferation (Fig. S2D). Notably, prolonged treatment with Dex-conditioned CAF medium resulted in significantly enlarged DU145 colonies (Fig. 4B) as well as DU145, CWR22Rv1, and LNCaP 3D spheroids (Fig. 4C). Furthermore, screening of Dex-conditioned CAF medium-treated LNCaP spheroids revealed elevated AR, KLK3, and c-Myc and reduced p21 mRNA expression, confirming a faster growing and more aggressive cell phenotype after GC treatment (Fig. 4D). We used commercially available pre-selected cytokine and chemokine protein arrays to identify soluble factors in the GC-conditioned CAF medium that might be responsible for the observed accelerated proliferation and to translate the already obtained mRNA data to the protein level. Treatment of primary isolated CAFs from four PCa patients with Dex for 3 d resulted in significantly altered cytokine and chemokine signatures (Fig. 4E, Fig. S2E). In addition to the general patient-specific heterogeneity in protein expression, CCL8, IL-8, and CXCL6 protein levels were significantly elevated and stable in all patient-derived primary CAFs after GC treatment. Additionally, to further validate the obtained results, we confirmed elevated and reduced IL-8 protein levels after treatment with either Dex or Pred alone or in combination with RU486 in PF179T-CAF, as well as in primary isolated NAFs and CAFs (Fig. 4F, Fig. S2F) using specific IL-8-coupled magnetic Luminex^®^ beads.

### IL-8 is a directly upregulated GR-target gene and is expressed predominantly in the TME after GC treatment

**Fig. 5:**
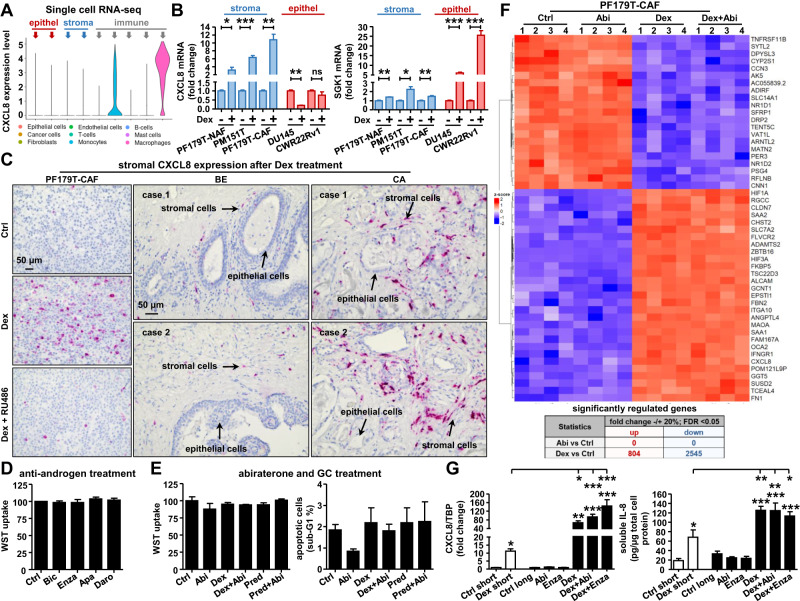
Prominent stromal IL-8 mRNA expression after GC incubation and unchanged cell viability of PF179T-CAF after treatment with NHTs. **A** CXCL8 mRNA expression levels were assessed in identified and selected cell subpopulations, analyzing single-cell RNA-seq datasets from 4 PCa patients. **B** CXCL8 and SGK1 mRNA expression was measured using PF179T-NAF, PF179T-CAF, PM151T, DU145, and CWR22Rv1 cells after 24 h 100 nM Dex treatment. mRNA data represent mean + SEM from 3 independent experiments (unpaired *t*-test; *P < 0.05; **P < 0.01; ***P < 0.001). **C** Representative CXCL8 specific in situ hybridization microscopy pictures of PF179T-CAF cells after 3 d treatment with 100 nM Dex alone or in combination with 6 µM RU486, as well as microscopy images of prominent stromal CXCL8 mRNA staining within benign and cancerous tissue sections after 3 d 100 nM Dex treatment. Magnification: 20x (scale bar = 50 µm). **D** Measurement of PF179T-CAF cell proliferation/viability after anti-androgen treatment with 2.5 µM Bic, 2.5 µM Enza, 2.5 µM Apa, or 2.5 µM Daro for 6 d. Data represent mean + SEM from 4 independent experiments (one-way ANOVA and correction for multiple testing using Dunnett’s comparison test). **E** Measurement of cell proliferation/viability and apoptosis of PF179T-CAF cells after single and combined treatment with 2.5 µM Abi, 100 nM Dex, or 100 nM Pred for 6 d. Data represent mean + SEM from 4 independent experiments (one-way ANOVA and correction for multiple testing using Dunnett’s comparison test). **F** bulk RNA-Seq analysis and heat map of top 50 significantly regulated genes of PF179T-CAF cells after single and combination treatment with 100 nM Dex and 2.5 µM Abi for 6 d, as well as assessment of general significantly up- and downregulated genes comparing bulk RNA-Seq datasets (2.5 µM Abi versus control, 100 nM Dex versus control) using chosen cut-offs (fold change +/-20%, FDR < 0.05). **G** IL-8 mRNA and protein expression after 3 d short-term 100 nM Dex treatment compared to IL-8 mRNA and protein expression in generated long-term treated PF179T-CAF cell sub-lines. mRNA and protein data represent mean + SEM from 3 independent experiments (one-way ANOVA and correction for multiple testing using Bonferroni’s comparison test; *P < 0.05; **P < 0.01; ***P < 0.001).

Since GCs and active GR signaling strongly influence IL-8 mRNA and protein expression, we tested whether IL-8 is a directly regulated GR-target. Screening publicly available ChIP-Seq datasets for putative GR-binding sites near or within the IL-8 gene revealed two predicted DNA-binding regions (R1 and R2) in several cell lines of different origins (Fig. 4G). Additionally, a time-course experiment demonstrated early IL-8 induction even after 2 h of Dex treatment (Fig. S2G). To verify the ChIP-Seq data predictions, GR-ChIP was performed using PF179T-CAF cells (Fig. 4H). Dex treatment resulted in a significantly elevated binding of activated GR to region R1, which RU486 blocked. Taken together, these results demonstrate that IL-8 is a direct GR-target gene. Surprisingly, screening for basal IL-8 expression in our scRNA-seq PCa datasets revealed only a strong detectable expression in immune cells (monocytes and macrophages), but not in the stromal or epithelial fraction (Fig. 5A), suggesting that IL-8 is specifically produced by the immune cell population in the prostate TME. Using immortalized PF179T-NAF, PM151T, PF179T-CAF, DU145, and CWR22Rv1 cells, detectable but very low basal IL-8 expression was confirmed in all screened prostate cell lines. Notably, increased IL-8 mRNA levels were observed only in stromal cell lines after Dex treatment, but not in epithelial cell lines, in contrast to the significantly induced epithelial SGK1 expression (Fig. 5B). Furthermore, prominent IL-8 mRNA expression was confirmed in PF179T-CAF cells by in situ hybridization in vitro and in the stroma of cultured benign and malignant prostate tissue pieces after 3 d of Dex treatment ex vivo (Fig. 5C, Fig. S3A).

### Standard androgen receptor pathway inhibitors (ARPIs) therapies have no impact on CAF survival

Next, we aimed to elucidate the functional impact of standard anti-androgen and abiraterone (Abi) treatments for the therapeutic targeting of CAFs. Interestingly, 6 d treatment with 2.5 µM (Fig. 5D) or 5 µM (Fig. S3B) bicalutamide (Bic), enzalutamide (Enza), apalutamide (Apa), or darolutamide (Daro) had no significant influence on cell proliferation/viability of treated PF179T-CAF. Furthermore, Abi alone or in combination with Dex or Pred led to similar results. No reduction in cell proliferation/viability, or elevated apoptosis was observed (Fig. 5E). As GCs are administered in combination with Abi, we further wanted to elucidate the possible impact of Abi on the general gene expression signature of PF179T-CAF by performing RNA-seq. Multivariate statistical analysis of RNA-seq data revealed no upregulated or downregulated genes after 6 d of Abi treatment, compared to 804 up- and 2545 downregulated genes upon Dex treatment under the selected cut-offs. Heat map visualization confirmed that Abi treatment alone or in combination with Dex did not affect the top 50 differentially expressed genes in PF179T-CAF cells (Fig. 5F). This result is unsurprising, as immortalized and primary isolated NAFs and CAFs have no detectable CYP17A1 mRNA or protein expression, the prime target of Abi, and a very low AR mRNA and protein expression (Fig. S4A). Accordingly, screening of IL-8 mRNA expression as an example gene of interest (GOI) resulted in no altered expression after a single Abi treatment compared to the significantly elevated expression after Dex or combined Dex+Abi treatment in PF179T-CAF cells (Fig. S4B). To ascertain that this observation was not a result of short-term treatment, we generated PF179T-CAF single Abi, Enza, Dex, and combined Enza+Dex and Abi+Dex long-term cultured and NHT-resistant cell sub-lines (Fig. S4C). No constricted cell growth was observed, but altered cell morphology was detected after long-term Dex, Abi+Dex, and Enza+Dex treatment (Fig. S4D). Notably, IL-8 mRNA and protein levels in the generated cell sub-lines were even higher after long-term Dex, Dex+Abi, and Dex+Enza treatments than after short-term treatment (Fig. 5G). Further screening of the selected upregulated chemokines (CXCL6 and CXCL1) and adhesion-related genes (ITGA10, FN1, CLDN7, and IRS2) (Fig. S4E) resulted in similar observations. Based on these results, we conclude that CAFs are not targetable by the standard anti-androgen or Abi therapy. Furthermore, these drugs seem to have no significant inhibitory effect on elevated GR-target gene expression after GC treatment.

### GC treatment results in a CAF cell morphology change

**Fig. 6:**
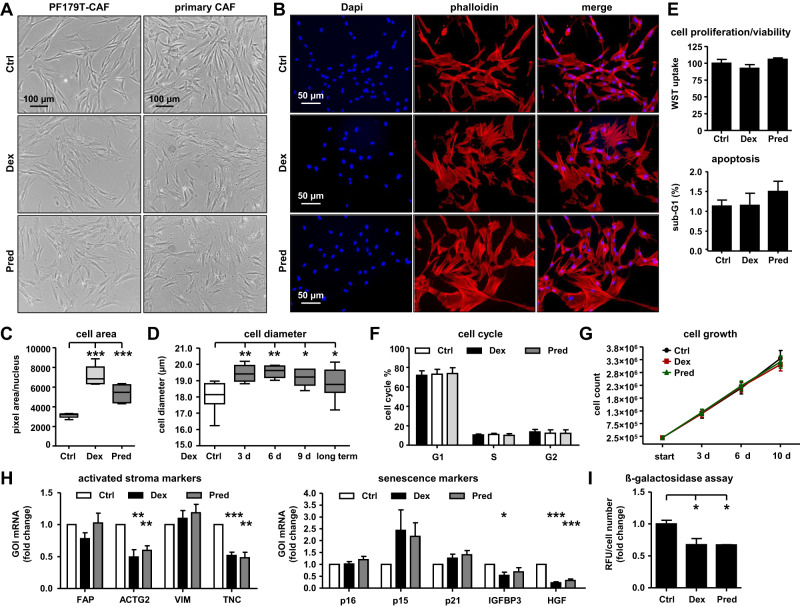
GC treatment results in a CAF cell morphology change. **A** Representative microscopy images for altered cell morphology of PF179T-CAF and primary isolated CAFs after 6 d 100 nM Dex or 100 nM Pred treatment. Magnification: ×10 (scale bar = 100 µm). **B** Representative IF microscopy images for altered actin filament structure within PF179T-CAF cells after 6 d 100 nM Dex or 100 nM Pred treatment. Magnification: 20× (scale bar = 50 µm). **C** Measurement of PF179T-CAF cell mean area after 6 d 100 nM Dex or 100 nM Pred treatment. Data represent mean + SEM from 3 independent experiments (one-way ANOVA and correction for multiple testing using Dunnett’s comparison test; ***P < 0.001) **D** Measurement of PF179T-CAF cell mean diameter changes after treatment with 100 nM Dex for 3 d, 6 d, 9 d, and after long-term cultivation with 100 nM Dex for over 30 passages. Data represent mean + SEM from 5 independent experiments (one-way ANOVA and correction for multiple testing using Dunnett’s comparison test; *P < 0.05; **P < 0.01). **E** Measurement of PF179T-CAF cell proliferation/viability and cell apoptosis after 6 d treatment with 100 nM Dex or 100 nM Pred. Data represent mean + SEM from 3 independent experiments (one-way ANOVA and correction for multiple testing using Dunnett’s comparison test; **P < 0.01). **F** Cell cycle measurement of PF179T-CAF cells after treatment with 100 nM Dex or 100 nM Pred for 6 d. Data represent mean + SEM from 3 independent experiments (one-way ANOVA and correction for multiple testing using Dunnett’s comparison test). **G** Assessment of PF179T-CAF cell growth during 3 d, 6 d, and 10 d cultivation with control (DMSO), or 100 nM Dex, or 100 nM Pred. Data represent mean + SEM from 3 independent experiments (one-way ANOVA and correction for multiple testing using Dunnett’s comparison test). **H** GOI mRNA expression measurement of selected activated stroma markers (FAP, ACTG2, VIM, and TNC) and selected senescence markers (p16, p15, p21, IGFBP3, and HGF) after 6 d treatment with 100 nM Dex or 100 nM Pred. Data represent mean + SEM from 3 independent experiments (one-way ANOVA and correction for multiple testing using Dunnett’s comparison test; *P < 0.05; **P < 0.01; ***P < 0.001). **I** Measurement of ß-galactosidase activity after 6 d treatment with 100 nM Dex or 100 nM Pred. Data represent mean + SEM from 3 independent experiments (one-way ANOVA and correction for multiple testing using Dunnett’s comparison test; *P < 0.05).

Based on the above-described effects on the cell morphology of Dex-treated PF179T-CAF cell sub-lines, we wanted to exclude cell line-specific artifacts due to long-term cultivation. Therefore, we treated PF179T-CAF and primary isolated CAFs with either Dex or Pred for 6 d and observed similar changes in cell morphology (Fig. 6A, B). Independent measurements of the CAF cell area and diameter confirmed significantly enlarged cells after GC treatment (Fig. 6C, D). Furthermore, we did not observe a significant change in cell proliferation/viability, apoptosis, cell cycle distribution, or long-term (up to 10 d) cell growth (Fig. 6E–G). Interestingly, by screening selected specific GOIs, which are known to be upregulated in activated or senescent fibroblasts (Fig. 6H), as well as by screening ß-galactosidase activity after GC treatment (Fig. 6I) we excluded the observed changes in cell morphology as a direct effect of differentiation in classically activated or senescent fibroblasts.

### Prolonged GC treatment is responsible for an altered fibroblast adhesion/ECM expression profile

**Fig. 7:**
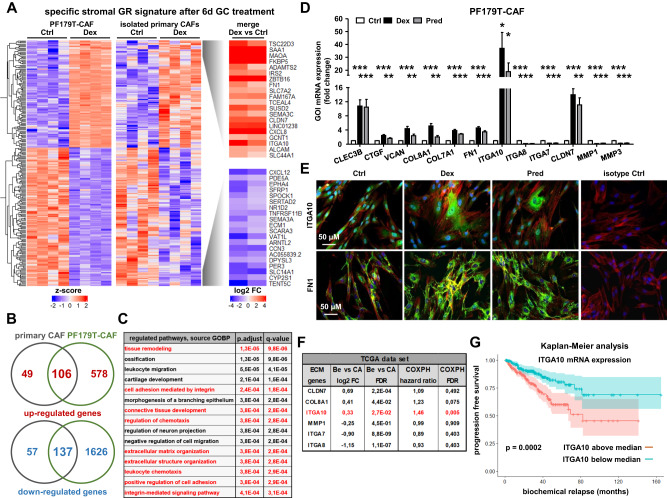
Prolonged GC treatment is responsible for ECM remodeling. **A** Bulk RNA-Seq analysis and heat map of top 40 significantly regulated genes using PF179T-CAF cells and primary isolated CAFs of 4 PCa patients, after DMSO (control) or 100 nM Dex treatment for 6 d. **B** Assessment of unique and commonly significantly up- and downregulated GR-target genes, comparing bulk RNA-Seq datasets from PF179T-CAF cells and primary isolated CAFs (fold change ±20%, FDR < 0.05). **C** GOBP pathway analysis for the identification of significantly regulated pathways after 6 d treatment with 100 nM Dex. **D** Confirmation of significantly altered GOI mRNA expression of selected ECM and adhesion markers (CLEC3B, CTGF, VCAN, COL8A1, COL7A1, FN1, ITGA10, ITGA8, ITGA7, CLDN7, MMP1, and MMP3) after 6 d treatment with 100 nM Dex or 100 nM Pred using PF179T-CAF cells. Data represent mean + SEM from at least 3 independent experiments (one-way ANOVA and correction for multiple testing using Dunnett’s comparison test; *P < 0.05; **P < 0.01; ***P < 0.001). **E** Representative IF microscopy images for altered ITGA10 and FN1 protein expression after 6 d 100 nM Dex or 100 nM Pred treatment using PF179T-CAF cells. Magnification: 20x (scale bar = 50 µm). **F** List of significantly up- or downregulated ECM genes after 6 d GC treatment, which expression corresponds to altered gene expression in primary PCa (TCGA dataset). **G** Kaplan–Meier analysis for ITGA10 mRNA expression using primary PCa patient data of the TCGA database.

To further investigate the observed novel CAF cell phenotype, we used our RNA-seq datasets representing PF179T-CAF and primary isolated CAFs after 6 d Dex treatment (Fig. 7A). The dataset comparisons resulted in 106 commonly up- and 137 downregulated genes between immortalized and primary isolated CAFs (Fig. 7B). Of note, follow-up gene ontology biological process (GOBP) pathway analyses revealed and confirmed not only a significantly regulated chemotaxis pathway, but also pathways associated with tissue remodeling, integrin-mediated cell adhesion, ECM- and ECM structure organization, and integrin-mediated signaling (Fig. 7C). Furthermore, we independently confirmed and extended the RNA-seq results with commercially available PCR gene arrays specifically selected for ECM and adhesion-related genes using PF179T-CAF (Fig. S5A). In the second step, we performed independent qRT-PCR analysis for selected GOIs, which were significantly upregulated or downregulated in immortalized as well as in primary isolated NAFs and CAFs after GC treatment (Fig. 7D, Fig. S5B). Moreover, elevated protein expression of the upregulated example GOIs, ITGA10, and FN1 after GC treatment was confirmed by IF staining (Fig. 7E). In the next step, a comparison analysis using the cancer genome atlas (TCGA) PCa datasets confirmed elevated CLDN7, COL8A1, and ITGA10 mRNA expression in PCa tissue samples. In contrast, MMP1, ITG7, and ITG8 mRNA expression levels were significantly reduced in primary PCa (Fig. 7F). Of note, using Kaplan-Meier analysis, elevated ITGA10 mRNA expression could be correlated with significantly earlier biochemical relapse and poor prognosis (Fig. 7G).

### Functional consequences of an altered adhesion/ECM expression profile for fibroblasts and epithelial cells

**Fig. 8:**
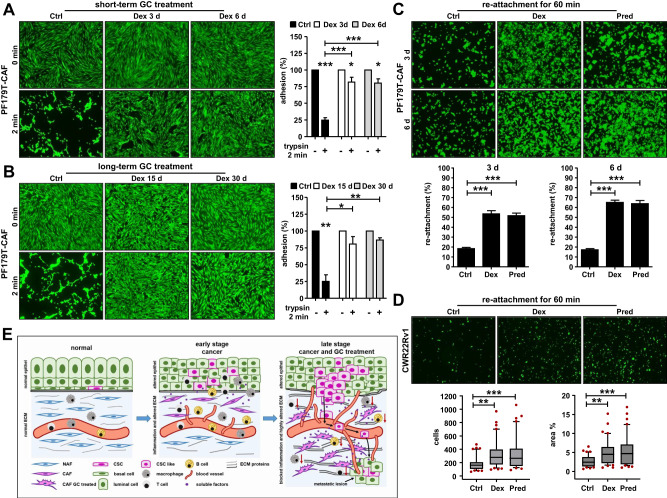
Functional consequences of ECM remodeling for stromal and epithelial cells. **A** Quantification of the adhesion capacity and representative microscopy images of PF179T-CAF cells after short-term culture with 100 nM Dex for 3 d and 6 d and pre-treatment with trypsin for 2 min before measurement. Data represent mean + SEM from 3 independent experiments (one-way ANOVA and correction for multiple testing using Bonferroni’s comparison test; *P < 0.05; ***P < 0.001). **B** Quantification of the adhesion capacity and representative microscopy images of PF179T-CAF cells after long-term culture with 100 nM Dex for 15 d and 30 d and pre-treatment with trypsin for 2 min before measurement. Data represent mean + SEM from 3 independent experiments (one-way ANOVA and correction for multiple testing using Bonferroni’s comparison test; *P < 0.05; **, *P* < 0.01). **C** Quantification of the re-attachment capacity and representative microscopy images of PF179T-CAF cells in a period of 60 min after treatment with 100 nM Dex or 100 nM Pred for 3 d and 6 d. Data represent mean + SEM from 3 independent experiments (one-way ANOVA and correction for multiple testing using Bonferroni’s comparison test; ***P < 0.001). **D** Quantification of the re-attachment capacity and representative microscopy images of CWR22Rv1 cells in a period of 60 min on a confluent PF179T-CAF cell monolayer. PF179T-CAF cells were pre-treated with 100 nM Dex or 100 nM Pred for 6 d before measurement. Data represent mean cell numbers + SEM from 3 independent experiments (one-way ANOVA and correction for multiple testing using Bonferroni’s comparison test; **P < 0.01; **P < 0.01; Box Whisker Plot with 10-90 percentile). **E** Schematic illustration of the proposed impact of altered soluble factor expression and ECM remodeling under the influence of GCs on tumor cell growth and disease progression based on the presented findings. The illustration was created in PowerPoint with the help of the MOTIFOLIO illustration tool kit.

GC treatment appears to significantly affect adhesion/ECM related genes and proteins. To evaluate possible functional consequences, we performed adhesion assays using PF179T-CAF cells. GC treatment for 3 d or 6 d resulted in re-enforced adhesion of Dex or Pred treated cells compared to DMSO treated control cells (Fig. 8A, Fig. S6A). Similar results were also observed after 6 d of GC treatment of PF179T-NAF cells (Fig. S6B). This phenomenon was also observed after long-term GC treatment (Fig. 8B). Moreover, ECM remodeling resulted in a faster re-attachment of GC-treated PF179T-CAF cells (Fig. 8C). Of note, also epithelial CWR22Rv1 cells re-attached faster to GC-treated CAFs compared to control cells (Fig. 8D), concluding that GC treatment not only has a significant impact on soluble factor expression in the TME but also results in a significant re-architecture of the ECM (Fig. 8E).

## Discussion

It is well recognized that elevated epithelial GR expression and activity have a major impact on the epithelial compartment, leading to accelerated tumor growth, therapy resistance, and decreased overall survival [3–5, 17, 18]. However, little is known about stromal GR expression and the direct GC-mediated effects on GR signaling in the prostate TME. Mohler et al. first observed heterogeneous epithelial and stromal GR expression in BPH and PCa tissue sections, demonstrating that GR expression was prominent in both epithelial BPH and stromal PCa cells but not in PCa epithelial cells [19]. Notably, in the present study, we confirmed and extended these findings at both the mRNA and protein levels. Re-analysis of our and publicly available scRNA-seq datasets comprising benign, BPH, and PCa tissues revealed a prominent cell type-specific GR mRNA expression profile in multiple identified stromal cell populations. Screening of primary PCa tissue samples and metastatic lesions confirmed intermediate or intense GR expression in the benign and tumor stroma, independent of the tumor stage. Moreover, a similar GR expression pattern was observed in immortalized fibroblasts, smooth muscle cells, and multiple primary isolated NAFs, and CAFs. Surprisingly, neither GR knockdown nor pharmacological GR inhibition as a single or combination treatment significantly affected CAF viability and apoptosis. Notably, NHT and abiraterone treatment did not affect CAF cell viability or CAF gene expression, indicating that CAFs cannot be directly targeted by ARPIs. Importantly, this finding is in contrast to previously published results for epithelial PCa cells [3–5]. However, control experiments confirmed a functional GR in used CAFs and short-term GC treatment resulted in an altered stromal GR-target gene signature. Our findings are supported by Hidalgo et al., who reported that PCa stroma exhibits altered GR-mediated transcriptional activity as well as an altered recruitment pattern of specific coactivators, thereby suggesting a role for the GC-modified TME in promoting hormone resistance in PCa [20]. After GC treatment, we identified a significantly altered stroma-specific chemokine expression profile at both mRNA and protein levels, which also had a significant functional impact on the epithelial compartment. Cultivation of multiple epithelial PCa cell models representing different disease and therapy stages, with medium from GC-treated CAFs resulted in accelerated cell proliferation in 2D cultures, elevated colony size, and 3D-spheroid growth. Our study highlights the up-regulation of CXCL6, IL-8, and CCL8 in response to GC treatment. These chemokines play significant roles in various stages of cancer development by facilitating angiogenesis, inflammation, and immune modulation [21]. CXCL6 is known to promote cell proliferation, angiogenesis, tumor growth, and metastasis in different malignancies, including PCa [22–25]. Recently, Wu et al. demonstrated that CXCL6 is involved in intercellular communication between prostate epithelial or cancer cells and fibroblasts [26]. IL-8 also enhances tumor cell proliferation, migration, invasion, angiogenesis, and metastasis in different malignancies, including PCa [27–33]. Elevated IL-8 levels have been associated with chemotherapy resistance in gastric and urothelial cancers and poor prognosis in PCa [34–36]. IL-8 signaling has also been shown to strongly influence epithelial to mesenchymal transition (EMT) in human carcinoma cells [37]. In PCa, IL-8 influences stromal cells and is a molecular determinant of androgen independence and progression [38, 39]. Furthermore, IL-8 is a master regulator of immune cells as it specifically recruits neutrophils to the TME [40]. High neutrophil count in the TME is also associated with PCa progression and patient survival [41, 42]. Additionally, CCL8 contributes to the immunosuppressive TME [43, 44]. Moreover, elevated CCL8 levels are associated with invasion and metastasis in various malignancies [45–47]. In summary, we found that an altered TME chemokine expression profile significantly affects the prostate epithelial compartment and promotes PCa cell growth. However, the authors would like to point out that the observed effects on elevated PCa cell proliferation cannot be attributed to an altered expression of a specific single soluble factor, but rather to a generally altered CAF secretome after GC treatment. We acknowledge several limitations of this study, including exclusion of the immune cell population. We speculate that GC-mediated elevated soluble factors may also have a significant impact on immune cells, specifically on the activity of tissue-associated macrophages (TAMs) and tissue-associated neutrophils (TANs) within the TME. However, studying these interactions was beyond the scope of this study, and remains to be done in the future.

In addition to the observed changes in the chemokine profile, GC treatment significantly influenced CAF cell morphology. Our findings were consistent with those reported by Ribeiro et al. [48]. They reported that Dex treatment of Wistar rats for 5 d resulted in morphological changes in the stromal cell population, suggesting fibroblast activation and smooth muscle cell atrophy. In our hands, the observed GC-mediated morphological changes did not interfere with normal cell growth and could not be attributed to differentiation into classical active or senescent fibroblasts. However, RNA-seq of GC-treated CAFs and subsequent pathway analysis revealed dramatic changes in the expression profiles of genes directly related to cellular adhesion and ECM organization. Remodeling of ECM is a key feature of cancer progression. In the context of GC-induced effects, our study highlights an increase in the expression of FN1 and ITGA10. FN1 plays a crucial role in PCa progression by promoting cancer cell adhesion, migration, invasion, and formation of the metastatic niche [49, 50]. The observed elevated FN1 expression was in concordance with the findings of Albrecht et al. After Dex treatment, they also observed induced FN1 expression, which directly affected cell proliferation [51]. ITGA10, on the other hand, is known to affect cell-ECM adhesion. It has been reported that ITGA10 can promote tumor growth and be successfully targeted by anti-ITGA10 antibodies in glioblastoma [52, 53]. Furthermore, ITGA10 can drive tumorigenesis in myxofibrosarcoma by promoting tumor cell survival through the activation of TRIO-RAC-RICTOR-mTOR signaling [54]. ITGTA10 is also associated with chemotherapy resistance in osteosarcoma patients by mediating activation of the PI3K/AKT pathway [55]. In line with these published results, we could also associate induced ITGA10 mRNA expression with significantly reduced progression-free survival of PCa patients. Furthermore, functional assays confirmed the induced cell adhesion capability of GC-treated NAFs and CAFs, with direct consequences on the attachment ability of epithelial PCa cells. In summary, preclinical data indicate that GC medication significantly affects fibroblasts in the TME with direct effects on altered soluble factor and adhesion/ECM protein expression, which results in accelerated PCa cell growth.

Generally, GCs are routinely administered as side medications owing to their anti-inflammatory and antiemetic effects in patients treated with taxane-based chemotherapy or abiraterone. Both therapy regimens are standard options for metastatic PCa, with an increasing impact due to the recent introduction of triplet therapy, but also because of the approval of PARP inhibitors in combination with abiraterone. However, the independent effect of GCs on survival is unclear, and GC administration has been associated with accelerated PCa progression and chemotherapy resistance in vitro and in vivo [56]. Zhang et al. demonstrated that GCs induce drug insensitivity in 89% of 157 examined established cell lines and patient-derived xenograft models. Notably, the recent findings by Zhao et al. are consistent with these observations. A post hoc analysis of the Phase III AFFIRM trial evaluating enzalutamide in CRPC provides clinical evidence that baseline GC use is associated with significantly reduced radiographic progression-free survival, time to PSA progression, and overall survival [57]. It was concluded that physicians should carefully consider whether the potential benefits of GC use outweigh the potential risks of treatment-related adverse events and impaired survival. Gained data on GC-mediated effects on the prostate TME and epithelial compartment within this preclinical study may explain the observed inferior clinical outcomes. Overall, accumulating preclinical and clinical data suggest that GC medication and elevated epithelial and stromal GR signaling can promote cancer progression and resistance to therapy. Thus, we propose clinical studies to assess the possibility of reducing or omitting the use of GCs during chemotherapy and abiraterone treatment, considering the resulting side effects of antineoplastic treatments. Furthermore, functional studies including the GR-AR network, GC-modified TME, and interaction with the immune system are necessary to uncover new therapeutic opportunities to overcome or prevent resistance and optimize PCa therapy in the future.

## Material and methods

### Cell culture and chemicals

PC3, DU145, LNCaP, and CWR22Rv1 cells were obtained from the American Type Culture Collection (ATCC, Rockville, MD, USA). LNCaPabl cells were generated after long-term cultivation of LNCaP cells in steroid-free medium [58]. LNCaPabl-Abi and LNCaPabl-Enza cells were generated after long-term abiraterone and enzalutamide treatments [59]. PC3-DR and DU145-DR cells were generated after long-term treatment with docetaxel [60]. PF179T-NAF, PF179T-CAF, PM151T, PC3, DU145, CWR22Rv1, PC3-DR, DU145-DR, LNCaP, LNCaPabl, LNCaPabl-Abi, and LNCaPabl-Enza cells were cultured as previously described [4, 7]. All cell lines were mycoplasma tested on a routine basis before cryo-conservation. The authenticity of all cell lines was validated by short tandem repeat (STR) profiling at the start of the project. Human primary prostatic normal (primary NAF) and cancer-associated fibroblasts (primary CAF) were established using an outgrowth method from prostate organoids from histologically verified benign and cancerous prostate regions from men undergoing radical prostatectomy (RPE). Fibroblasts were maintained in DMEM (Cat# BE12-707F; Lonza, Cologne, Germany) containing 10% fetal bovine serum (FBS; Supreme, Cat# P30-3031; THP Medical Products, Vienna, Austria), 1% penicillin/streptomycin (Cat# 17-602E, Lonza), and 2% GlutaMAX™ (Cat# 35050-038; GIBCO, Fisher Scientific, Vienna, Austria) and used until passage 15 or lower.

### Chemicals

The following chemicals were used at concentrations indicated in the results and figure legends: dimethylsulfoxide (DMSO; Cat# D2650, Sigma Aldrich, Vienna, Austria), doxycycline (Dox; Cat# D9891, Sigma Aldrich), mifepristone (RU486; Cat# S2606), dexamethasone (Dex; Cat# S1322), prednisolone (Pred; Cat# S2570), apalutamide (Apa; Cat# S2840) (Selleck Chemicals, Munich, Germany), enzalutamide (Enza; Cat# HY-70002), abiraterone (Abi; Cat# HY-75054) (Hycultech, Beutelsbach, Germany), darolutamide (Daro; Cat# HY-16985), bicalutamide (Bic; Cat# HY-14249) (MedChemExpress, Stockholm, Sweden), docetaxel (Doc; Cat# MCE-HY-B0011), and cabazitaxel (Cab; Cat# MCE-HY-15459) (THP Medical Products).

### Patient material

Patients were selected from the Innsbruck Uro-Biobank. The use of archived materials was approved by the Ethics Committee of the Medical University of Innsbruck (EV 1072/2018). Written consent was obtained from all patients and documented in the database of the University Hospital Innsbruck in agreement with statutory provisions. Tissue microarrays (TMAs) containing benign and primary cancer tissue cores from PCa patients who underwent open retropubic or robot-assisted RPE and metastatic lesions were used to evaluate GR expression. Matched benign samples were excised from histologically confirmed nonmalignant regions of the RPE specimens. For ex vivo tissue cultures as well as for primary NAF/CAF isolation and culturing, the use of primary material was approved by the Ethics Committee of the Medical University of Innsbruck (EV 4837). Written consent was obtained from all patients and documented in the database of the University Hospital Innsbruck in agreement with statutory provisions. As previously described, benign and cancerous tissue samples were obtained from explanted prostates and further processed after positive quality control [7].

### Immunohistochemistry (IHC)

GR IHC was performed on a Ventana BenchMark device (Roche, Vienna, Austria) using an anti-GR rabbit mAb (D6H2L; Cat# 12041) antibody (1:200; Cell Signaling, Danvers, MA, USA). The IHC specificity of the GR antibody has been confirmed previously [4]. Images were taken with a TissueFAXS imaging system using a Zeiss Imager Z2 microscope equipped with a Pixelink PL‐D674CU-CYL-07451 camera and processed using the TissueFAXS software version 7.137 (Tissue Gnostics, Vienna, Austria). TMA images were evaluated using the modified “quick-score” protocol: staining intensity was scored 0–4 (0 = absent, 1 = weak, 2 = intermediate, and 3 = strong). The percentage of positively stained cells was scored 0-4 (0 = absent, 1 *≤* 10%, 2 *≤* 50%, 3 *≤* 75%, 4 *≥* 75%). Both scores were multiplied to obtain an immune-reactivity score (IRS) ranging from 0 to 12.

### In situ hybridization (ISH)

Tissue samples were cultured in the presence of 100 nM Dex for 3 d. For probe specificity testing, PF179T-CAF cells were cultured in the presence of DMSO, 100 nM Dex, or 100 nM Dex + 6 µM RU486 for 3 d. Cells were harvested and resuspended in a buffer containing 450 µl citrate plasma and 11.3 µl 1 M calcium chloride in a 15 ml vial. 45 µl thrombin 120 NIH-U/ml (Cat# T4648-1KU; Sigma Aldrich) was added until the cell suspension coagulated. The cell coagulates and harvested tissue pieces were transferred to prepared biopsy histosettes, formalin-fixed, paraffin-embedded, and processed for in situ hybridization. IL-8 (CXCL8) ISH was performed using a specific RNAscope Hs-IL-8 probe (Cat# 310381; Advanced Cell Diagnostics, Milan, Italy) and the RNAscope 2.5 HD Red kit (Cat# 322350; Advanced Cell Diagnostics) according to the manufacturer’s instructions. Images were taken with a TissueFAXS imaging system using a Zeiss Imager Z2 microscope equipped with a Pixelink PL‐D674CU-CYL-07451 camera and processed using the TissueFAXS software version 7.137 (Tissue Gnostics). ISH pictures were quantified using the Fiji open-source platform for biological-image analysis [61].

### Statistical analysis

GraphPad Prism 9.4.1 (RRID:SCR_002798) (Dotmatics, Boston, USA) was used for statistical analyses. The Gaussian distribution was determined for all experiments using Kolmogorov-Smirnov and D’Agostino & Pearson omnibus normality tests. Differences between treatment groups were analyzed using unpaired or paired Student’s *t*-test or Mann–Whitney *U* test, depending on Gaussian distribution. Multiple treatment groups were compared using one-way ANOVA and corrected for multiple testing using Bonferroni or Dunn’s multiple comparison test method. *P* values < 0.05 were considered significant. All differences highlighted by asterisks are statistically significant, as encoded in the figure legends (*P < 0.05, **P < 0.01, ***P < 0.001). Data are presented as the mean + Standard Error of Mean (SEM) unless otherwise specified.

### Supplementary information

Figure S1

Figure S2

Figure S3

Figure S4

Figure S5

Figure S6

Supplemental Material

Supplementary Information

## Data Availability

All Affymetrix array and bulk RNA-seq data generated or analyzed during this study are included in this published article, and its supplementary information files are available in the NCBI Gene Expression Omnibus (GEO) repository, https://www.ncbi.nlm.nih.gov/gds. Single-cell RNA-seq data are available as a public resource for data exploration using the Unicle web tool (https://unicle.life/portals/).

## References

[1] Li Y, Chan SC, Brand LJ, Hwang TH, Silverstein KA, Dehm SM (2013). Androgen receptor splice variants mediate enzalutamide resistance in castration-resistant prostate cancer cell lines. Cancer Res.

[2] Hu R, Lu C, Mostaghel EA, Yegnasubramanian S, Gurel M, Tannahill C (2012). Distinct transcriptional programs mediated by the ligand-dependent full-length androgen receptor and its splice variants in castration-resistant prostate cancer. Cancer Res.

[3] Arora VK, Schenkein E, Murali R, Subudhi SK, Wongvipat J, Balbas MD (2013). Glucocorticoid receptor confers resistance to antiandrogens by bypassing androgen receptor blockade. Cell.

[4] Puhr M, Hoefer J, Eigentler A, Ploner C, Handle F, Schaefer G (2018). The glucocorticoid receptor is a key player for prostate cancer cell survival and a target for improved antiandrogen therapy. Clin Cancer Res.

[5] Kroon J, Puhr M, Buijs JT, van der Horst G, Hemmer DM, Marijt KA (2016). Glucocorticoid receptor antagonism reverts docetaxel resistance in human prostate cancer. Endocr Relat Cancer.

[6] Shah N, Wang P, Wongvipat J, Karthaus WR, Abida W, Armenia J (2017). Regulation of the glucocorticoid receptor via a BET-dependent enhancer drives antiandrogen resistance in prostate cancer. Elife.

[7] Puhr M, Eigentler A, Handle F, Hackl H, Ploner C, Heidegger I (2021). Targeting the glucocorticoid receptor signature gene Mono Amine Oxidase-A enhances the efficacy of chemo- and anti-androgen therapy in advanced prostate cancer. Oncogene.

[8] Zhang Z, Zhou C, Li X, Barnes SD, Deng S, Hoover E (2020). Loss of CHD1 promotes heterogeneous mechanisms of resistance to AR-targeted therapy via chromatin dysregulation. Cancer Cell.

[9] Sottnik JL, Zhang J, Macoska JA, Keller ET (2011). The PCa tumor microenvironment. Cancer Microenviron.

[10] Ippolito L, Morandi A, Taddei ML, Parri M, Comito G, Iscaro A (2019). Cancer-associated fibroblasts promote prostate cancer malignancy via metabolic rewiring and mitochondrial transfer. Oncogene.

[11] Li D, Xu W, Chang Y, Xiao Y, He Y, Ren S (2023). Advances in landscape and related therapeutic targets of the prostate tumor microenvironment. Acta Biochim Biophys Sin.

[12] Tannock IF, de Wit R, Berry WR, Horti J, Pluzanska A, Chi KN (2004). Docetaxel plus prednisone or mitoxantrone plus prednisone for advanced prostate cancer. N Engl J Med.

[13] Heidenreich A, Bracarda S, Mason M, Ozen H, Sengelov L, Van Oort I (2014). Safety of cabazitaxel in senior adults with metastatic castration-resistant prostate cancer: results of the European compassionate-use programme. Eur J Cancer.

[14] Small EJ, Lance RS, Gardner TA, Karsh LI, Fong L, McCoy C (2015). A randomized phase II trial of sipuleucel-T with concurrent versus sequential abiraterone acetate plus prednisone in metastatic castration-resistant prostate cancer. Clin Cancer Res.

[15] Heidegger I, Fotakis G, Offermann A, Goveia J, Daum S, Salcher S (2022). Comprehensive characterization of the prostate tumor microenvironment identifies CXCR4/CXCL12 crosstalk as a novel antiangiogenic therapeutic target in prostate cancer. Mol Cancer.

[16] Joseph DB, Henry GH, Malewska A, Iqbal NS, Ruetten HM, Turco AE (2020). Urethral luminal epithelia are castration-insensitive cells of the proximal prostate. Prostate.

[17] Xie N, Cheng H, Lin D, Liu L, Yang O, Jia L (2015). The expression of glucocorticoid receptor is negatively regulated by active androgen receptor signaling in prostate tumors. Int J Cancer.

[18] Pak S, Suh J, Park SY, Kim Y, Cho YM, Ahn H (2022). Glucocorticoid receptor and androgen receptor-targeting therapy in patients with castration-resistant prostate cancer. Front Oncol.

[19] Mohler JL, Chen Y, Hamil K, Hall SH, Cidlowski JA, Wilson EM (1996). Androgen and glucocorticoid receptors in the stroma and epithelium of prostatic hyperplasia and carcinoma. Clin Cancer Res.

[20] Hidalgo AA, Montecinos VP, Paredes R, Godoy AS, McNerney EM, Tovar H (2011). Biochemical characterization of nuclear receptors for vitamin D3 and glucocorticoids in prostate stroma cell microenvironment. Biochem Biophys Res Commun.

[21] Nagarsheth N, Wicha MS, Zou W (2017). Chemokines in the cancer microenvironment and their relevance in cancer immunotherapy. Nat Rev Immunol.

[22] Song M, He J, Pan QZ, Yang J, Zhao J, Zhang YJ (2021). Cancer-associated fibroblast-mediated cellular crosstalk supports hepatocellular carcinoma progression. Hepatology.

[23] Li J, Tang Z, Wang H, Wu W, Zhou F, Ke H (2018). CXCL6 promotes non-small cell lung cancer cell survival and metastasis via down-regulation of miR-515-5p. Biomed Pharmacother.

[24] Karagiannis GS, Saraon P, Jarvi KA, Diamandis EP (2014). Proteomic signatures of angiogenesis in androgen-independent prostate cancer. Prostate.

[25] Begley LA, Kasina S, MacDonald J, Macoska JA (2008). The inflammatory microenvironment of the aging prostate facilitates cellular proliferation and hypertrophy. Cytokine.

[26] Wu Y, Clark KC, Niranjan B, Chueh AC, Horvath LG, Taylor RA (2023). Integrative characterisation of secreted factors involved in intercellular communication between prostate epithelial or cancer cells and fibroblasts. Mol Oncol.

[27] Ning Y, Manegold PC, Hong YK, Zhang W, Pohl A, Lurje G (2011). Interleukin-8 is associated with proliferation, migration, angiogenesis and chemosensitivity in vitro and in vivo in colon cancer cell line models. Int J Cancer.

[28] Milovanovic J, Todorovic-Rakovic N, Radulovic M (2019). Interleukin-6 and interleukin-8 serum levels in prognosis of hormone-dependent breast cancer. Cytokine.

[29] Reis ST, Leite KR, Piovesan LF, Pontes-Junior J, Viana NI, Abe DK (2012). Increased expression of MMP-9 and IL-8 are correlated with poor prognosis of Bladder Cancer. BMC Urol.

[30] An H, Zhu Y, Xie H, Liu Y, Liu W, Fu Q (2016). Increased expression of interleukin-8 is an independent indicator of poor prognosis in clear-cell renal cell carcinoma. Tumour Biol.

[31] MacManus CF, Pettigrew J, Seaton A, Wilson C, Maxwell PJ, Berlingeri S (2007). Interleukin-8 signaling promotes translational regulation of cyclin D in androgen-independent prostate cancer cells. Mol Cancer Res.

[32] Maynard JP, Ertunc O, Kulac I, Baena-Del Valle JA, De Marzo AM, Sfanos KS (2020). IL8 expression is associated with prostate cancer aggressiveness and androgen receptor loss in primary and metastatic prostate cancer. Mol Cancer Res.

[33] Cioni B, Nevedomskaya E, Melis MHM, van Burgsteden J, Stelloo S, Hodel E (2018). Loss of androgen receptor signaling in prostate cancer-associated fibroblasts (CAFs) promotes CCL2- and CXCL8-mediated cancer cell migration. Mol Oncol.

[34] Zhai J, Shen J, Xie G, Wu J, He M, Gao L (2019). Cancer-associated fibroblasts-derived IL-8 mediates resistance to cisplatin in human gastric cancer. Cancer Lett.

[35] Kikuchi H, Maishi N, Annan DA, Alam MT, Dawood RIH, Sato M (2020). Chemotherapy-induced IL8 upregulates MDR1/ABCB1 in tumor blood vessels and results in unfavorable outcome. Cancer Res.

[36] Sharma J, Gray KP, Harshman LC, Evan C, Nakabayashi M, Fichorova R (2014). Elevated IL-8, TNF-alpha, and MCP-1 in men with metastatic prostate cancer starting androgen-deprivation therapy (ADT) are associated with shorter time to castration-resistance and overall survival. Prostate.

[37] Fernando RI, Castillo MD, Litzinger M, Hamilton DH, Palena C (2011). IL-8 signaling plays a critical role in the epithelial-mesenchymal transition of human carcinoma cells. Cancer Res.

[38] Araki S, Omori Y, Lyn D, Singh RK, Meinbach DM, Sandman Y (2007). Interleukin-8 is a molecular determinant of androgen independence and progression in prostate cancer. Cancer Res.

[39] Yuan A, Chen JJ, Yao PL, Yang PC (2005). The role of interleukin-8 in cancer cells and microenvironment interaction. Front Biosci.

[40] Zippoli M, Ruocco A, Novelli R, Rocchio F, Miscione MS, Allegretti M (2022). The role of extracellular vesicles and interleukin-8 in regulating and mediating neutrophil-dependent cancer drug resistance. Front Oncol.

[41] Bahig H, Taussky D, Delouya G, Nadiri A, Gagnon-Jacques A, Bodson-Clermont P (2015). Neutrophil count is associated with survival in localized prostate cancer. BMC Cancer.

[42] Wang Y, Dong X, Qu Z, Peng K, Sun X, Chen R (2020). Correlation between peripheral blood neutrophil-lymphocyte ratio and CD34 expression in prostate cancer. BMC Cancer.

[43] Kobayashi H, Gieniec KA, Lannagan TRM, Wang T, Asai N, Mizutani Y (2022). The origin and contribution of cancer-associated fibroblasts in colorectal carcinogenesis. Gastroenterology.

[44] Zhang Y, Lazarus J, Steele NG, Yan W, Lee HJ, Nwosu ZC (2020). Regulatory T-cell depletion alters the tumor microenvironment and accelerates pancreatic carcinogenesis. Cancer Discov.

[45] Kim ES, Nam SM, Song HK, Lee S, Kim K, Lim HK (2021). CCL8 mediates crosstalk between endothelial colony forming cells and triple-negative breast cancer cells through IL-8, aggravating invasion and tumorigenicity. Oncogene.

[46] Farmaki E, Chatzistamou I, Kaza V, Kiaris H (2016). A CCL8 gradient drives breast cancer cell dissemination. Oncogene.

[47] Torres S, Bartolome RA, Mendes M, Barderas R, Fernandez-Acenero MJ, Pelaez-Garcia A (2013). Proteome profiling of cancer-associated fibroblasts identifies novel proinflammatory signatures and prognostic markers for colorectal cancer. Clin Cancer Res.

[48] Ribeiro DL, Rafacho A, Bosqueiro JR, Taboga SR, Goes RM (2008). Cellular changes in the prostatic stroma of glucocorticoid-treated rats. Cell Tissue Res.

[49] Erdogan B, Ao M, White LM, Means AL, Brewer BM, Yang L (2017). Cancer-associated fibroblasts promote directional cancer cell migration by aligning fibronectin. J Cell Biol.

[50] Petrini I, Barachini S, Carnicelli V, Galimberti S, Modeo L, Boni R (2017). ED-B fibronectin expression is a marker of epithelial-mesenchymal transition in translational oncology. Oncotarget.

[51] Albrecht M, Janssen M, Konrad L, Renneberg H, Aumuller G (2002). Effects of dexamethasone on proliferation of and fibronectin synthesis by human primary prostatic stromal cells in vitro. Andrologia.

[52] Munksgaard Thoren M, Chmielarska Masoumi K, Krona C, Huang X, Kundu S, Schmidt L (2019). Integrin alpha10, a novel therapeutic target in glioblastoma, regulates cell migration, proliferation, and survival. Cancers.

[53] Masoumi KC, Huang X, Sime W, Mirkov A, Munksgaard Thoren M, Massoumi R (2021). Integrin alpha10-antibodies reduce glioblastoma tumor growth and cell migration. Cancers.

[54] Okada T, Lee AY, Qin LX, Agaram N, Mimae T, Shen Y (2016). Integrin-alpha10 dependency identifies RAC and RICTOR as therapeutic targets in high-grade myxofibrosarcoma. Cancer Discov.

[55] Li H, Shen X, Ma M, Liu W, Yang W, Wang P (2021). ZIP10 drives osteosarcoma proliferation and chemoresistance through ITGA10-mediated activation of the PI3K/AKT pathway. J Exp Clin Cancer Res.

[56] Zhang C, Wenger T, Mattern J, Ilea S, Frey C, Gutwein P (2007). Clinical and mechanistic aspects of glucocorticoid-induced chemotherapy resistance in the majority of solid tumors. Cancer Biol Ther.

[57] Zhao JL, Fizazi K, Saad F, Chi KN, Taplin ME, Sternberg CN (2022). The effect of corticosteroids on prostate cancer outcome following treatment with enzalutamide: a multivariate analysis of the phase III AFFIRM trial. Clin Cancer Res.

[58] Culig Z, Hoffmann J, Erdel M, Eder IE, Hobisch A, Hittmair A (1999). Switch from antagonist to agonist of the androgen receptor bicalutamide is associated with prostate tumour progression in a new model system. Br J Cancer.

[59] Hoefer J, Akbor M, Handle F, Ofer P, Puhr M, Parson W (2016). Critical role of androgen receptor level in prostate cancer cell resistance to new generation antiandrogen enzalutamide. Oncotarget.

[60] Puhr M, Hoefer J, Schäfer G, Erb HH, Oh SJ, Klocker H (2012). Epithelial-to-mesenchymal transition leads to docetaxel resistance in prostate cancer and is mediated by reduced expression of miR-200c and miR-205. Am J Pathol.

[61] Schindelin J, Arganda-Carreras I, Frise E, Kaynig V, Longair M, Pietzsch T (2012). Fiji: an open-source platform for biological-image analysis. Nat Methods.

