# Supplementary figures and images for: Glucocorticoid treatment influences prostate cancer cell growth and the tumor microenvironment via altered glucocorticoid receptor signaling in prostate fibroblasts
Source: Oncogene. 2023 Nov 29;43(4):235–47. doi: 10.1038/s41388-023-02901-5 (PMC10798901; doi:10.1038/s41388-023-02901-5)

Figure S1

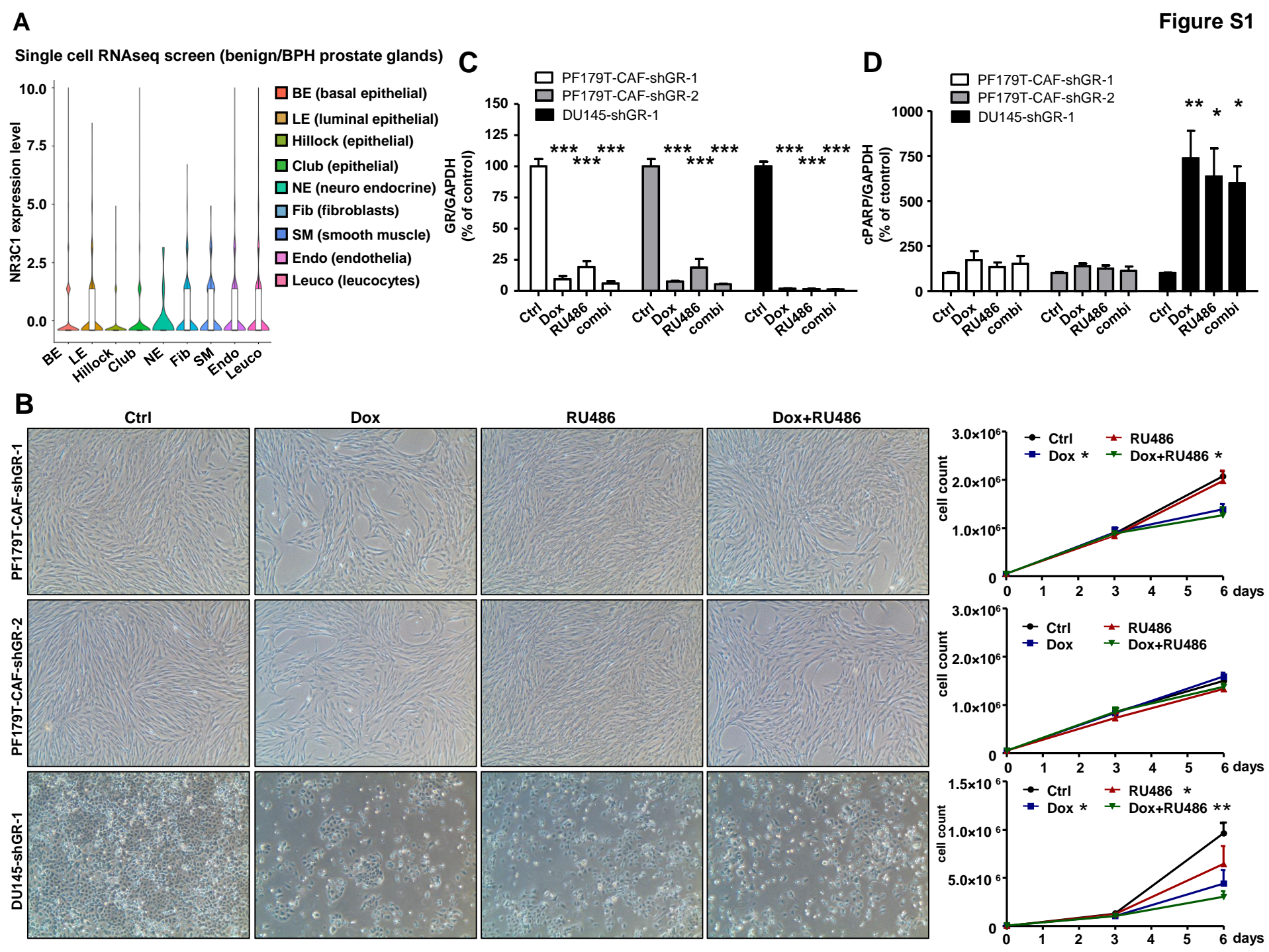

Supplement: Supplementary file 1 — Figure S1 [file 41388_2023_2901_MOESM1_ESM.pdf]

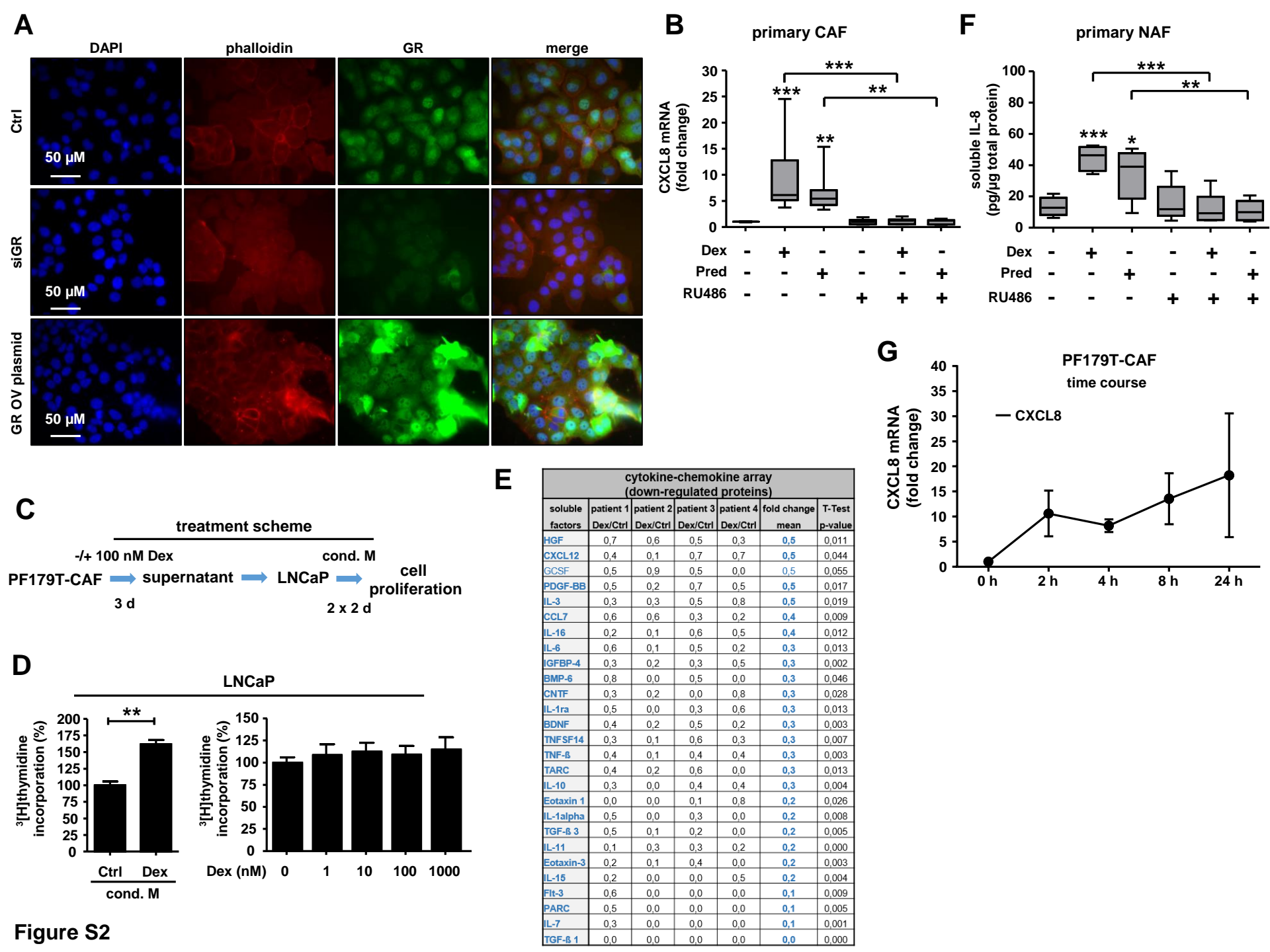

Figure S2

Supplement: Supplementary file 2 — Figure S2 [file 41388_2023_2901_MOESM2_ESM.pdf]

Figure S3

A

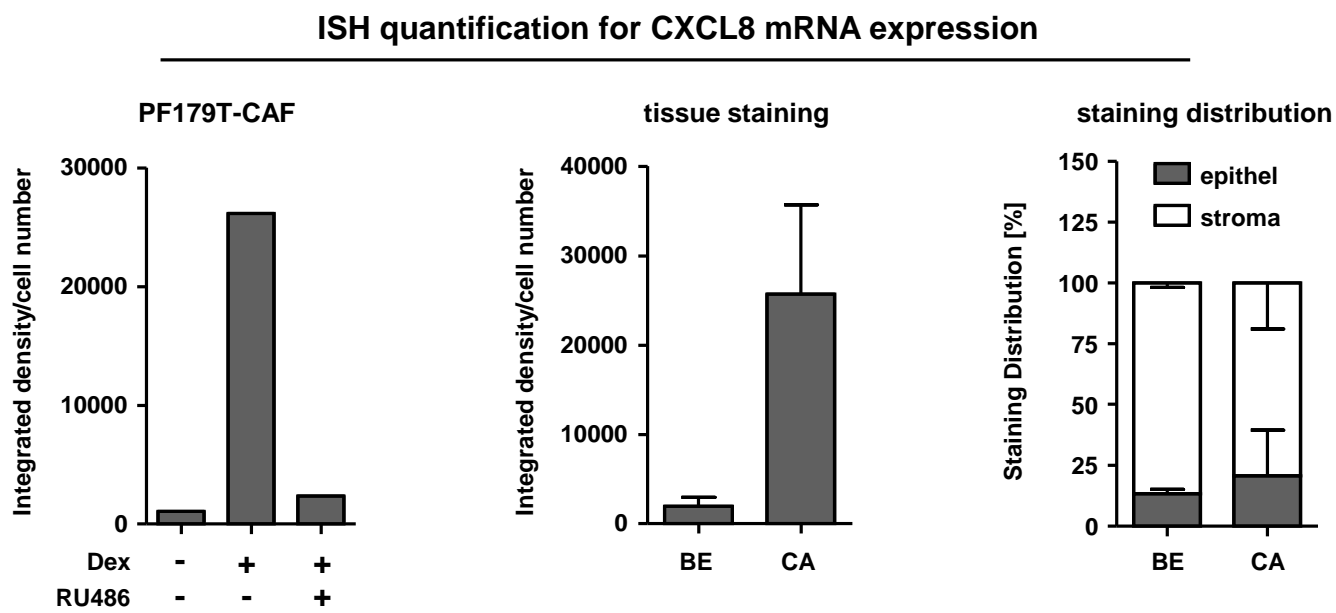

B

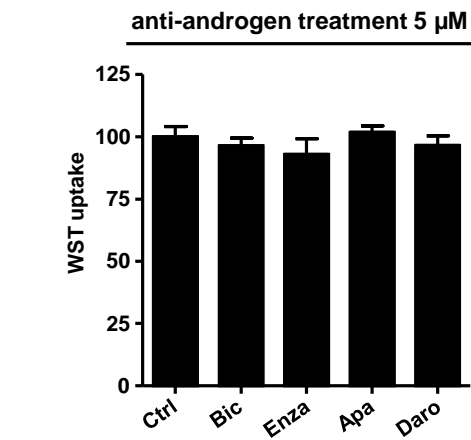

Supplement: Supplementary file 3 — Figure S3 [file 41388_2023_2901_MOESM3_ESM.pdf]

Figure S4

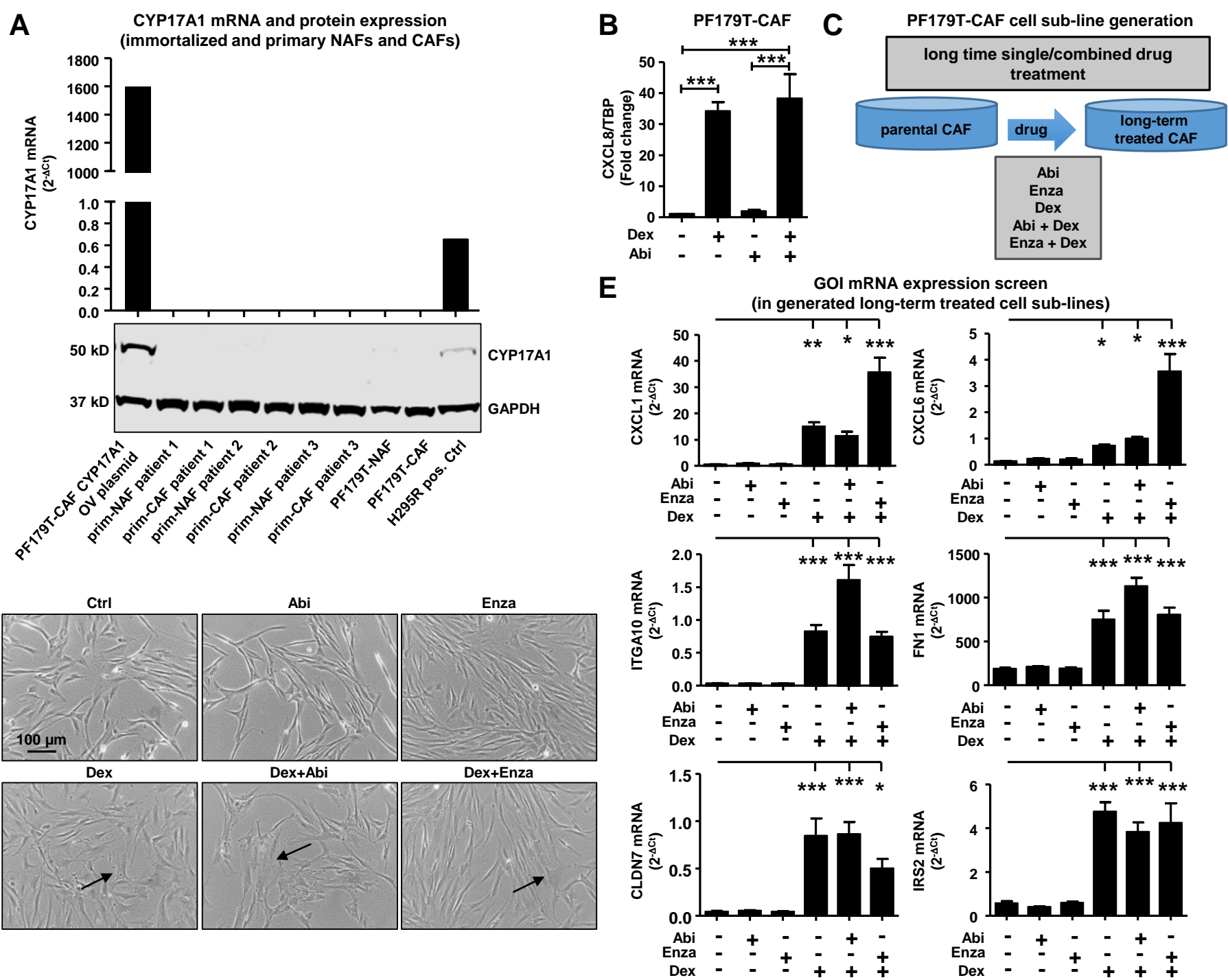

Supplement: Supplementary file 4 — Figure S4 [file 41388_2023_2901_MOESM4_ESM.pdf]

Figure S6

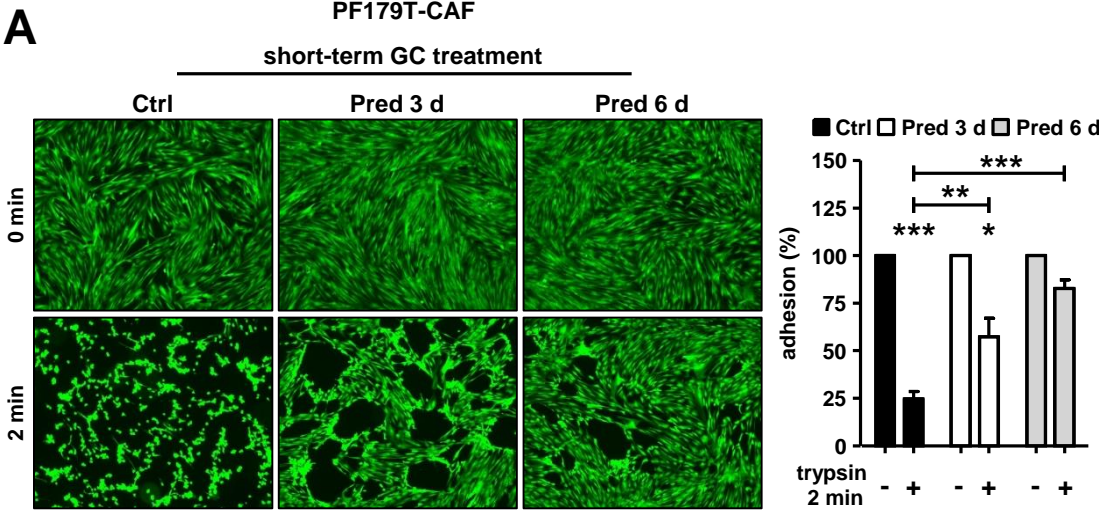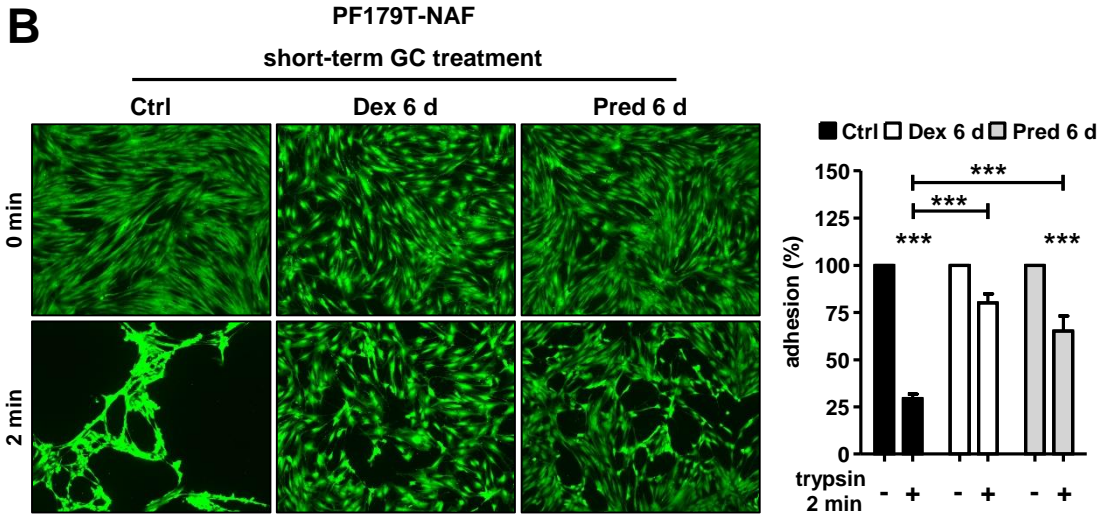

Supplement: Supplementary file 6 — Figure S6 [file 41388_2023_2901_MOESM6_ESM.pdf]
