# Supplementary material for: Glucocorticoid treatment influences prostate cancer cell growth and the tumor microenvironment via altered glucocorticoid receptor signaling in prostate fibroblasts
Source: Oncogene. 2023 Nov 29;43(4):235–47. doi: 10.1038/s41388-023-02901-5 (PMC10798901; doi:10.1038/s41388-023-02901-5)

Figure S5

A

PCR gene array for ECM and adhesion genes

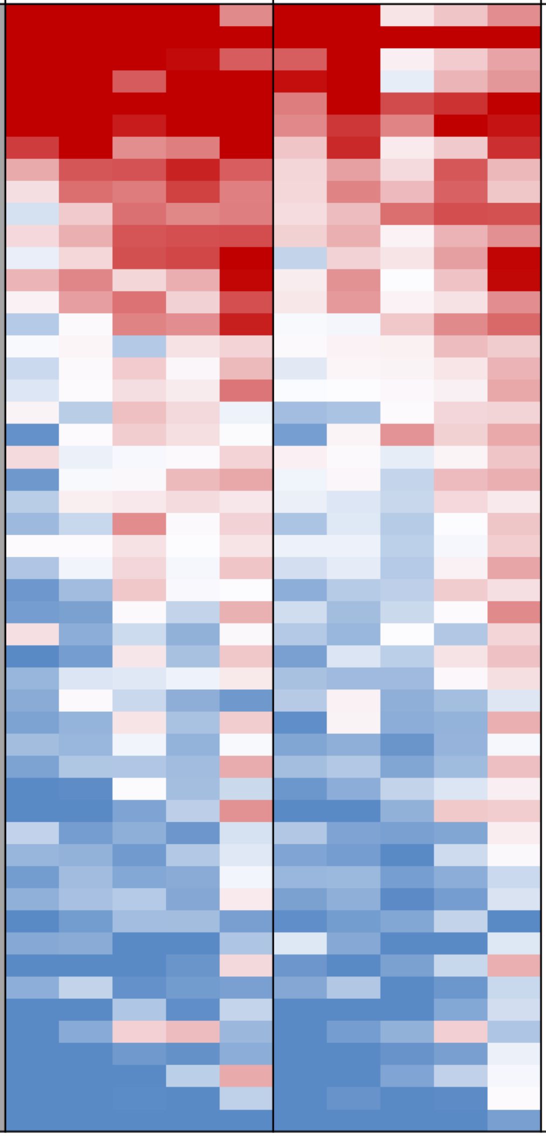

genes	Dex+Pred fold change	statistics T-Test p-value
SPP1	22,5	0,3236
CLEC3B	8,1	0,0025
VCAN	4,0	0,1522
COL8A1	3,6	0,0614
FN1	3,3	0,0007
COL7A1	3,2	0,0173
CTGF	2,0	0,0629
COL5A1	1,8	0,1791
COL1A1	1,6	0,1483
LAMA2	1,6	0,1629
ITGA5	1,5	0,1494
ITGB4	1,4	0,5249
ADAMTS13	1,3	0,4871
CDH1	1,2	0,4773
ADAMTS1	1,2	0,5866
COL6A2	0,8	0,4440
CTNND1	0,8	0,0204
ITGA1	0,8	0,0299
TGFBI	0,8	0,1825
SGCE	0,8	0,1685
COL12A1	0,8	0,0151
ITGB3	0,8	0,1471
SPG7	0,8	0,1614
LAMB1	0,7	0,1266
CTNNA1	0,7	0,0002
ITGAV	0,7	0,0015
MMP2	0,6	0,0936
VCAM1	0,6	0,0013
CD44	0,6	0,0029
MMP11	0,6	0,0206
TIMP2	0,6	0,0000
THBS2	0,5	0,0462
MMP16	0,5	0,0022
ECM1	0,4	0,0001
ICAM1	0,4	0,0001
LAMA3	0,4	0,0006
ADAMTS8	0,4	0,0179
ITGA4	0,4	0,0000
TIMP1	0,4	0,0000
ITGA2	0,4	0,0001
LAMA1	0,3	0,0018
TIMP3	0,3	0,0007
ITGA6	0,3	0,0000
VTN	0,3	0,0000
TNC	0,3	0,0004
MMP3	0,3	0,0003
MMP10	0,3	0,1423
ITGA8	0,2	0,0000
ITGB2	0,2	0,0007
ITGA7	0,2	0,0000
MMP1	0,1	0,0008

B

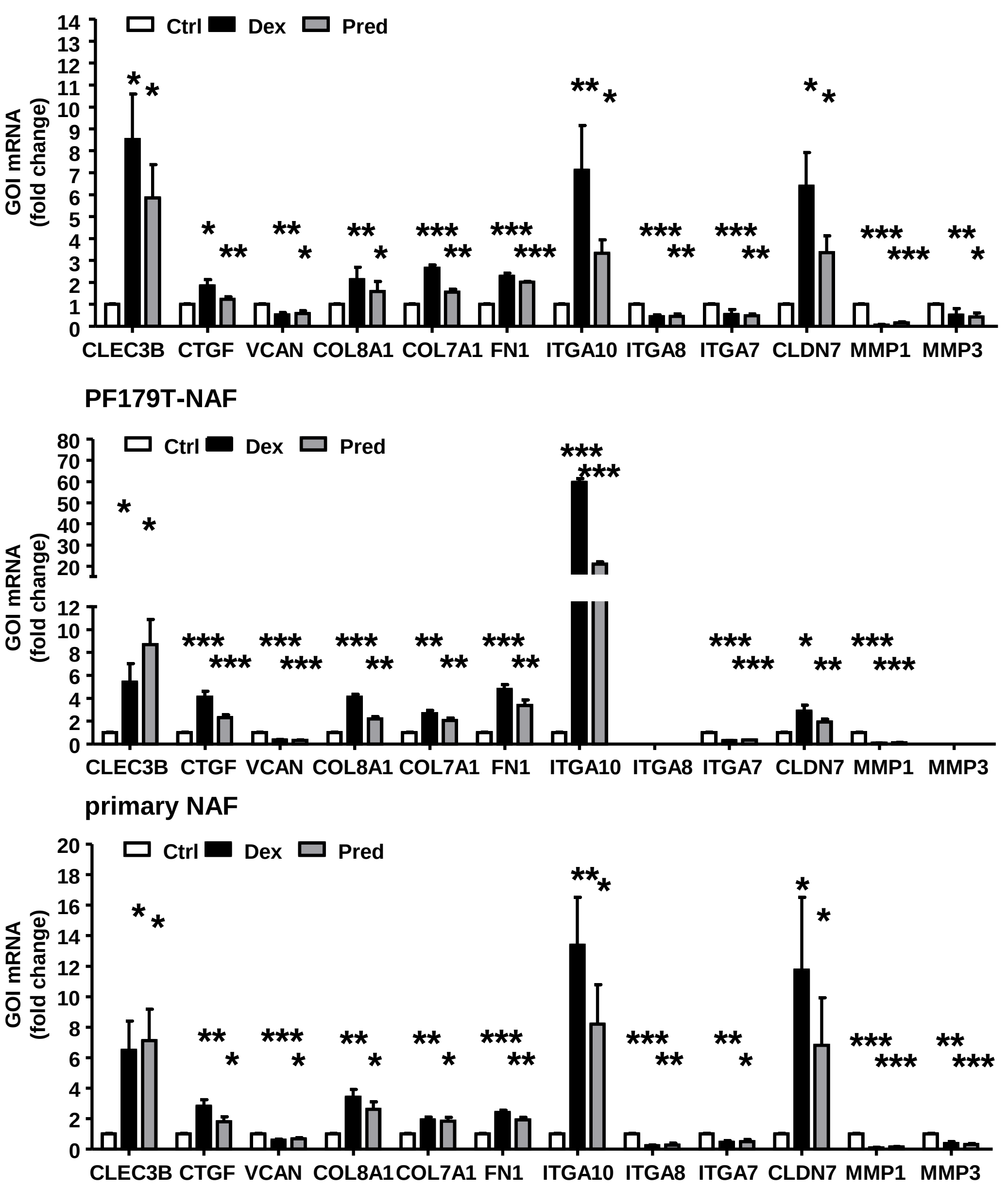

Supplement: Supplementary file 5 — Figure S5 [file 41388_2023_2901_MOESM5_ESM.pdf]
