# Supplementary material for: Glucocorticoid treatment influences prostate cancer cell growth and the tumor microenvironment via altered glucocorticoid receptor signaling in prostate fibroblasts
Source: Oncogene. 2023 Nov 29;43(4):235–47. doi: 10.1038/s41388-023-02901-5 (PMC10798901; doi:10.1038/s41388-023-02901-5)
Supplement: Supplementary file 7 — Supplemental Material [file 41388_2023_2901_MOESM7_ESM.docx]

**Additional File 1 Supplementary Materials & Methods**

**RNA isolation, cDNA synthesis and qRT-PCR:** Total RNA from cell lines was isolated with the Blirt TOTAL RNA KIT (Cat# 66.EM09-250; LabConsulting, Vienna, Austria) according to the manufacturer's instructions. RNA yield and quality were determined on a Nanodrop2000 system (ThermoFisher Scientific, Vienna, Austria). cDNA synthesis was done with a Luna Script RT Super Mix Kit (Cat# M3010X; New England Biolabs, Ipswich, USA) and qRT-PCR was performed on an ABI PRISM 7500-FAST system (ThermoFisher Scientific) using a Luna Universal Probe qPCR Master Mix (Cat# M3004X; New England Biolabs) according to the manufacturer`s protocols. All expression data were normalized to TATA-box binding protein (TBP). Custom primers were used at a concentration of 800 nmol/L each and FAM-TAMRA labeled probes at 150 nmol/L. Primer sequences for TBP, AR, KLK3 were previously described ([1](#_ENREF_1)). TaqMan gene-expression assays (ThermoFisher Scientific) were used for NR3C1 (Hs00353740_m1), GILZ (Hs00608272_m1), SGK1 (Hs00985033_g1), IGFBP3 (Hs00426289_m1), IGFBP1 (Hs00236877_m1), CXCL8/IL-8 (Hs00174103_m1), c-Myc (Hs00153408_m1), p21/CDKN1A (Hs99999142_m1), p16/CDKN2A (Hs00923894_m1), p15/CDKN2B (Hs00793225_m1), HGF (Hs00300159_m1), FAP (Hs00990806_m1), SMA/ACTG2 (Hs01123712_m1), Vim (Hs00185584_m1), TNC (Hs01115665_m1), CLEC3B (Hs00162844_m1), VCAN (Hs00171642_m1), COL8A1 (Hs00156669_m1), COL7A1 (Hs00164310_m1), CTGF (Hs00170014_m1), FN1 (Hs01549976_m1), ITGA10 (Hs01006910_m1), CLDN7 (Hs00600772_m1), ITGA8 (Hs00233321_m1), ITGA7 (Hs01056475_m1), MMP1 (Hs00899658_m1), MMP3 (Hs00968305_m1). For assessment of GR target genes associated with the ECM and cell adhesion after treatment with 100 nM Dex or 100 nM Pred for 6 d, commercially available TaqMan® Array Human Extracellular Matrix & Adhesion Molecules 96-well plates (Cat# 4418778) were used according to the manufacturer`s protocol.

**western blot:** Cells were lysed in LDS sample buffer, 50 μg total protein was separated on NuPAGE Novex 4%–12% Bis-Tris Protein Gels (Cat# NP0329BOX; ThermoFisher Scientific) and transferred to 0.2 μm nitrocellulose membranes (Cat# 10600001; GE Healthcare, Vienna, Austria). The following antibodies were used: anti-GR rabbit Mab (Cat# 12041, RRID:AB_2631286) (1:1000; Cell Signaling), anti-AR rabbit Mab (Cat# 40065, RRID:AB_2799166) (1:1000; Cell Signaling), anti-GAPDH (Cat# MAB374, RRID:AB_2107445) (1:50.000; Millipore, Vienna, Austria), anti-cPARP (Cat# G7341, RRID:AB_430876) (1:500; Promega, Vienna, Austria), and anti-CYP17A1 (Cat# TA503442) (1:1000; Origene, Rovkville, USA).

**transient transfections:** For CYP17A1 over-expression experiments, cells were transfected with a CYP17A1 over-expression plasmid (NM_000102 Human cDNA Clone) (Origene, Herford, Germany) and Lipofectamine2000 (Cat# 10696153; ThermoFisher Scientific) for 3 d according to the manufacturer`s protocols.

**soluble protein profiling:** Primary CAFs isolated from 4 PCa patients were cultured in the absence or presence of 100 nM Dex for 3 d. After 3 days, supernatant was collected, centrifuged, filtered, and stored at -80 °C for further use. Supernatant was loaded onto RayBio® Human Cytokine Antibody Array G-Series 1000 (G6, G7; RayBiotech, Norcross, GA) which facilitates the detection of 120 cytokines and chemokines. Array plates were processed according to the manufacturer’s instructions. Relative fluorescent intensity of spots was scanned with the GenePixx 4000B microarray scanner (Molecular Devices, San Jose, USA) and specific signal intensities at 532 nm were normalized to background of used standard CAF medium. Soluble IL-8 levels in the supernatant of PF179T-NAF, PF179T-CAF, as well as in primary isolated NAFs and CAFs were assessed using specific Human IL-8/CXCL8 High Sensitivity Luminex Performance Assays (LHSCM208) (R&D Systems, Vienna, Austria) according to the manufacturer’s instructions.

**proliferation & viability measurements:** Proliferation was assessed using ^3^[H]thymidine incorporation (Cat# MT-6032; Hanke Laboratory Products, Vienna, Austria) and cell viability/proliferation was determined using the CellTiter 96® AQueous one solution assay (Cat# G3580; Promega) according to the manufacturer`s protocol. Briefly, 1.5 x 10^3^ cells/96-well PC3, PC3-DR, DU145, DU145-DR, CWR22Rv1, 1 x 10^4^ cells/96-well LNCaP, LNCaPabl, LNCaPabl-Enza, and LNCaPabl-Abi were seeded in 96-well plates and incubated for specific time points. Treatment with the indicated drugs was performed on the next day. Measurements were done in at least 3 independent biological experiments with at least 3 technical replicates.

**apoptosis & cell cycle distribution:** Cells were trypsinized and pellets were re-suspended in propidium iodide (PI) buffer (0.2% Triton-X-100, 2 ng/mL Na-Citrate, and 0.05 mg/mL PI) and kept light-protected at 4°C for 1 h. The percentage of apoptotic cells (sub-G1), as well as cell cycle distribution have been determined using FACS Calibur (Becton Dickinson, Heidelberg, Germany).

**cell growth, cell diameter and cell area measurements:** For cell growth experiments 2.5 x 10^5^ PF179T-CAF cells were seeded in T25 flasks and treated with 100 nM Dex or 100 nM Pred. At indicated days, cell numbers were determined by a CASY cell counter (Schärfe System, Reutlingen, Germany). 2.5 x 10^5^ cells were re-seeded, and treated again with specific drugs. At day 10, cells were finally counted. Cell diameter was assessed at indicated days using a CASY cell counter. For cell area assessment, taken IF pictures were analyzed (pixel area/nucli) after 6 days, using the CellProfiler 4.2.4 software ([2](#_ENREF_2)).

**ß-Galactosidase activity:** 2.5 x 10^5^ PF179T-CAF cells were seeded in T25 cell culture flasks. On the following day, medium was exchanged. Cells were cultured in the presence of ethanol (Ctrl), 100 nM Dex or 100 nM Pred for 6 days. Medium was changed and renewed after 3 d. Cells were harvested, cell number was determined using a CASY counter, and the resulting pellets were stored at -80°C for further usage. The ß-galactosidase activity was assessed using a commercially available Senescence ß-Galactosidase Activity Assay (Cat# 23833, Cell Signaling). Fluorescence was measured with a fluorescence plate reader (Agilent BioTek Cytation 5, reader, ThermoFisher Scientific) set with excitation at 360 nm and emission at 465 nm within 30 minutes of adding the stop solution.

**3D-spheroid assay:**  7.5 x 10^3^  (DU145, CWR22Rv1) or 1 x 10^4^ (LNCaP) cells were suspended in a final volume of 40 µl medium/well (conditioned Ctrl or 100 nM Dex PF179T-CAF) and cultured for 8 d using Perfecta3D® 96 well hanging drop plates (Sigma). On day 4, 20 µl old medium was replaced by 30 µl of fresh medium. Spheroid formation was assessed at day 8. Cell spheroids were imaged on a JuLI^TM^ fluorescence microscope (NanoEntek, Seoul, Korea). Spheroid size was evaluated using the free software ImageJ (RRID:SCR_003070) (U.S. National Institutes of Health, Bethesda, MD, USA). Spheroid size was determined using the formula for the geometric *mean radius= ½(a x b)^1/2^*, where a and b are the two orthogonal diameters of the spheroid as described in detail elsewhere ([3](#_ENREF_3), [4](#_ENREF_4)).

**colony formation assay:** 1 x 10^3^ cells were seeded into a 75 cm^2^ cell culture flasks with 12 ml Ctrl (DMSO) or 100 nM Dex treated conditioned medium and incubated for 12 d. Medium change was performed at day 6. Subsequently, the cell colonies were fixed with 100% ice-cold methanol for 5 min and stained with PBS containing 20% methanol and 0.5% crystal violet (Sigma) for 2 min. Colony numbers and colony size were determined and quantified using the free software ImageJ (RRID:SCR_003070).

**adhesion and re-attachment assays:** 3 x 10^5^ (for 6 d) or 6 x 10^5^ (for 3 d) PF179T-CAF and PF179T-NAF cells were seeded in T25 flasks and cultured in the presence of either Ctrl, or 100 nM Dex, or 100 nM Pred for 3 and 6 d. Confluent cells were stained with calcein (#Enz-52002, ENZO Life Science, Lausen, Switzerland) for 30 min at 37°C and treated with 1 ml trypsin for exact 2 min. After 2 min the reaction was stopped with 4 ml medium. Medium and floating cells were removed gently and 2 ml new medium was added. Cell culture pictures were taken and the green area was analyzed and quantified using the free software ImageJ (RRID:SCR_003070). For re-attachment experiments using PF179T-CAF, cells were pre-treated with either Ctrl, or 100 nM Dex, or 100 nM Pred for 3 and 6 days. After indicated days, cells were harvested and counted. 1 x 10^5^ cells were seeded in 48 well plates, cultured for 60 min, stained with calcein, and additionally cultured for 30 min at 37°C. After incubation, plates were washed gently with PBS refilled with 500 µl medium and pictures were acquired. Green area was analyzed and quantified using the free software ImageJ (RRID:SCR_003070). For re-attachment experiments using CWR22Rv1-GFP cells, PF179T-CAF cells were seeded in 48 well plates and were pre-treated with either Ctrl, or 100 nM Dex, or 100 nM Pred for 3 and 6 d. 1 x 10^5^ CWR22Rv1-GFP cells were added to the confluent PF179T-CAF cell layer within the 48 well plates and cultured for 60 min. After incubation, plates were washed gently with PBS refilled with 500 µl medium and pictures were acquired. Green cells and green area were analyzed and quantified using the free software ImageJ (RRID:SCR_003070).

**Immunofluorescence** **(IF):** For visualization of GR translocation, cells were seeded onto glass coverslips and allowed to attach for 48 h. Cells were cultured in the absence or presence of 100 nM Dex for 60 min. Subsequently, the cells were washed with PBS and fixed with 4% paraformaldehyde (PFA) for 10 min. Cells were washed with PBS and permeabilized with PBS / 1% BSA / 0.2% Triton X100 for 5 min. After a 30 min blocking step with PBS / 1% BSA, coverslips were incubated for 1 h with the primary antibody anti-GR rabbit Mab (Cat# 12041, RRID:AB_2631286) (1:75; Cell Signaling). After washing, coverslips were incubated with the following fluorescence-labeled secondary antibody goat anti-rabbit 488 (Cat# A-11070) (1:500, ThermoFisher Scientific) and with rhodamine phalloidin reagent (Cat# ab235138) (1:1.000, Abcam) according to the manufacturer`s protocol. Coverslips were finally washed and mounted with Vectashield Hard Set mounting medium containing DAPI (Cat# H-1500; Vector Laboratories, Burlingame, CA) on glass slides. The cells were visualized using fluorescent microscopy on a Zeiss Axio Imager M1 microscope.

After treatment with 100 nM Dex and 100 nM Pred for 6 days, cells were fixed with 3 % PFA for 15 min and permeabilized with 0.5 % TritonX-100. After blocking with 1 % bovine serum albumin (BSA) for 1 h at room temperature cells were stained with rhodamin phalloidin (1:1000, Abcam) and incubated for 3 h at 37 °C. Afterwards, cells were washed with PBS and blocked again for 1 h with 5 % goat serum (Agilent, Vienna, Austria) containing 0,3 % TritonX-100. After washing with PBS, cells were stained with anti-FN1-antibody (Cat# 26836) (1:200, Cell Signaling), or anti-ITGA10-antibody (Cat# AB6030) (1:100, Sigma) and rabbit isotype control (Cat# 3900S) [1:50, rabbit (DA1E) mAb IgG XP isotype control, Cell Signaling], incubated overnight at 4 °C and detected with anti-rabbit-Alexa Fluor 488 antibody. Nuclei were stained with Hoechst33342 (1 μg/ml, Sigma). Image acquisition was performed with Celena S Digital Imaging System (Logos Biosystems, Gyeonggi-do, South Korea).

**generation of lentiviral vectors & plasmid construction:** Construction of a Dox inducible short hairpin RNA (shRNA) vector against human GR (shGR-1, shGR-2) was done as already described ([1](#_ENREF_1)). GR knockdown in the established PF179T-CAF-shGR-1/shGR-2 and DU145-shGR-1 cell sub-lines was achieved by adding 1 µg/ml Dox for indicated time points.

**screening of GR binding sites:** For the identification of potential GR binding sides near the CXCL8 gene, GR binding elements were extracted using publicly available ChIP-seq datasets GSE79431, GSE79803, GSE85343, GSE39879 and GSE51497 from the GEO database ([5-8](#_ENREF_5)) and visualized with the IGV browser.

**Chromatin Immunoprecipitation (ChIP):** 4 x 10^6^ PF179T-CAF cells were seeded in 15 cm^2^ cell culture dishes and grown for 48 h. Specific treatment with DMSO, 100 nM Dex, or 100 nM Dex and 6 µM RU-486 was performed for 16 h. Chromatin IP was performed with the SimpleChIP® Plus Enzymatic Chromatin Kit (Magnetic Beads) (Cat# 9005S; Cell Signaling) according to the manufacturer´s protocol. Positive control Histone H3 (D2B12, Cat# 4620) XP® rabbit mAb (Cell Signaling), negative control normal rabbit IgG (Cat# 2729, Cell Signaling) or GR (D6H2L, Cat# 12041) XP® Rabbit mAb (Cell Signaling) were added to the IP samples and incubated for 16 h at 4°C. Elution of chromatin from antibody/protein G magnetic beads as well as reversal of cross links was done according to the kit protocol. Quantification of DNA was done by qRT-PCR on an ABI PRISM 7500-FAST system (ThermoFisher Scientific) using specific primers and probes for 2 identified GR-CXCL8 binding sides: R1-fwd, 5´-TAT.AAA.AAG.CCA.CCG.GAG.CA-3´; R1-rev, 5´-GCC.AGC.TTG.GAA.GTC.ATG.TT-3´; R1-probe, 5´-CAG.CAG.AGC.ACA.CAA.GCT.TC-3´; R2-fwd, 5´-TGT.CCT.CCA.CAG.AAT.GTT.GG-3´; R2-rev, 5´-TTT.GCA.AAT.ATG.CTT.AGG.CTT.T-3´; R2-probe, 5´-GCG.CTT.TAG.CAT.AGC.TGG.AC-3´ and for the internal positive control Beta-2-microglobulin (B2M), ChIP-B2M fwd, 5´-TGC TGT CTC CAT GTT TGA TGT ATC T-3´; ChIP-B2M-rev, 5´-TCT CTG CTC CCC ACC TCT AAG T-3´; ChIP-B2M-probe, 5´-CAG GTT GCT CCA CAG GTA GCT CTA G-3´.

**bioinformatic analysis of microarray datasets:** 5 x 10^6^ primary CAFs from 4 different PCa patients were seeded into T175 tissue flasks. On the next day specific treatments were performed as followed. Group 1: vehicle Ctrl was treated with DMSO for 24 h. Group 2: 100 nM Dex for 24h. Group 3: 100 nM Dex and 12 µM RU486 treatment for 24 h. After 24 h cells were harvested and total RNA was isolated with the Blirt EXTRACTME TOTAL RNA KIT (LabConsulting). Total RNA was isolated and Affymetrix microarray analyses using Clariom^TM^ S human arrays for were performed by a commercial service provider (Eurofins/Aros, Aarhus, Denmark) according to standard procedures. Published PF179T-CAF-shGR1 datasets ([9](#_ENREF_9)) (GSE150432) have been re-analyzed and combined with primary CAF datasets. The experimental data of primary CAFs have been deposited in the NCBI Gene Expression Omnibus (GEO) (RRID:SCR_005012) (GSE240480). Analyses were performed on gene level (filtered for Refseq mRNA annotation), differentially expressed genes were identified using moderated t-tests (R package *limma*), and p-values were adjusted for multiple testing based on the false discovery rate according to the Benjamini-Hochberg procedure. Genes with more than two-fold change at a FDR<0.1 (or >1.5 fold change and p<0.01 where stated) were considered as significantly differentially expressed. Gene set enrichment analysis (RRID:SCR_003199) on log2 fold change pre-ranked data was performed using GSEA software (RRID:SCR_005724) and gene sets for biological hallmark processes and pathways (MSigDB) as well as defined GR gene signatures. Heatmaps were generated using Genesis 1.8.1.

**single cell RNA-seq datasets:** Public available single cell RNAseq data from normal and BPH human prostates were used to characterize cell type specific GR mRNA expression ([10](#_ENREF_10), [11](#_ENREF_11)). Single cell RNAseq datasets from PCa patients were re-analyzed to characterize cell type specific GR and IL-8 mRNA expression in different cell sub-populations ([12](#_ENREF_12)).

**bulk RNA-seq datasets**

Total cellular RNA-extraction was performed with the Blirt EXTRACTME TOTAL RNA KIT (LabConsulting). Quality control, poly(A) enrichment, strand specific library preparation and sequencing (paired-end 150 bp, >20 million read pairs per sample) was performed by Novogene. Analysis of the data was performed in R. Read alignment (gencode release 35, GRCh38.p13) and counting was performed with the Rsubread package. Differential gene expression analysis was performed with the limma package (RRID:SCR_010943) using the voom/treat pipeline. GOBP over representation analysis was performed with the clusterProfiler package (RRID:SCR_016884). The experimental data have been deposited in the NCBI Gene Expression Omnibus (GEO) (RRID:SCR_005012) (GSE240659).

1. Puhr M, Hoefer J, Eigentler A, Ploner C, Handle F, Schaefer G, et al. The Glucocorticoid Receptor Is a Key Player for Prostate Cancer Cell Survival and a Target for Improved Antiandrogen Therapy. Clin Cancer Res. 2018;24(4):927-38.

2. McQuin C, Goodman A, Chernyshev V, Kamentsky L, Cimini BA, Karhohs KW, et al. CellProfiler 3.0: Next-generation image processing for biology. PLoS Biol. 2018;16(7):e2005970.

3. Zhou Y, Arai T, Horiguchi Y, Ino K, Matsue T, Shiku H. Multiparameter analyses of three-dimensionally cultured tumor spheroids based on respiratory activity and comprehensive gene expression profiles. Anal Biochem. 2013;439(2):187-93.

4. Kelm JM, Timmins NE, Brown CJ, Fussenegger M, Nielsen LK. Method for generation of homogeneous multicellular tumor spheroids applicable to a wide variety of cell types. Biotechnol Bioeng. 2003;83(2):173-80.

5. Arora VK, Schenkein E, Murali R, Subudhi SK, Wongvipat J, Balbas MD, et al. Glucocorticoid receptor confers resistance to antiandrogens by bypassing androgen receptor blockade. Cell. 2013;155(6):1309-22.

6. Kadiyala V, Sasse SK, Altonsy MO, Berman R, Chu HW, Phang TL, et al. Cistrome-based Cooperation between Airway Epithelial Glucocorticoid Receptor and NF-kappaB Orchestrates Anti-inflammatory Effects. J Biol Chem. 2016;291(24):12673-87.

7. Nakamoto M, Ishihara K, Watanabe T, Hirosue A, Hino S, Shinohara M, et al. The Glucocorticoid Receptor Regulates the ANGPTL4 Gene in a CTCF-Mediated Chromatin Context in Human Hepatic Cells. PLoS One. 2017;12(1):e0169225.

8. Sahu B, Laakso M, Pihlajamaa P, Ovaska K, Sinielnikov I, Hautaniemi S, et al. FoxA1 specifies unique androgen and glucocorticoid receptor binding events in prostate cancer cells. Cancer Res. 2013;73(5):1570-80.

9. Puhr M, Eigentler A, Handle F, Hackl H, Ploner C, Heidegger I, et al. Targeting the glucocorticoid receptor signature gene Mono Amine Oxidase-A enhances the efficacy of chemo- and anti-androgen therapy in advanced prostate cancer. Oncogene. 2021;40(17):3087-100.

10. Henry GH, Malewska A, Joseph DB, Malladi VS, Lee J, Torrealba J, et al. A Cellular Anatomy of the Normal Adult Human Prostate and Prostatic Urethra. Cell Rep. 2018;25(12):3530-42 e5.

11. Joseph DB, Henry GH, Malewska A, Iqbal NS, Ruetten HM, Turco AE, et al. Urethral luminal epithelia are castration-insensitive cells of the proximal prostate. Prostate. 2020;80(11):872-84.

12. Heidegger I, Fotakis G, Offermann A, Goveia J, Daum S, Salcher S, et al. Comprehensive characterization of the prostate tumor microenvironment identifies CXCR4/CXCL12 crosstalk as a novel antiangiogenic therapeutic target in prostate cancer. Mol Cancer. 2022;21(1):132.
