# Supplementary material for: Glucocorticoid treatment influences prostate cancer cell growth and the tumor microenvironment via altered glucocorticoid receptor signaling in prostate fibroblasts
Source: Oncogene. 2023 Nov 29;43(4):235–47. doi: 10.1038/s41388-023-02901-5 (PMC10798901; doi:10.1038/s41388-023-02901-5)
Supplement: Supplementary file 8 — Supplementary Information [file 41388_2023_2901_MOESM8_ESM.docx]

**Supplementary information:**

**additional file 1.** Supplementary M&M

**Supplementary Figure Legends:**

**Figure S1**:

**(A)** Assessment of GR mRNA expression levels within identified and isolated cell sub-populations of benign and BPH prostates, using publicly available scRNA-seq datasets. **(B)** Measurement of PF179T-CAF-shGR-1/shGR-2 and DU145-shGR-1 cell growth and representative cell culture microscopy pictures, after single inducible GR knockdown with 1 µg/ml Dox, pharmacological inhibition with 6 µM RU486 or combination treatment for 6 d. Data represent mean + SEM from 3 independent experiments (unpaired t-test; *, P< 0.05). **(C)** Normalization and quantification of specific GR and cPARP **(D)** Western blot bands, after single inducible GR knockdown with 1 µg/ml Dox, pharmacological inhibition with 6 µM RU486 or combination treatment for 6 d, using PF179T-CAF-shGR-1/shGR-2 and DU145-shGR-1 cells. Data represent mean + SEM from at least 3 independent experiments (one-way ANOVA and correction for multiple testing using Dunnett's comparison test; *, P< 0.05; **, P< 0.01; ***, P< 0.001).

**Figure S2:**

**(A)** GR antibody specificity test for IF usage. DU145 cells were transfected with either siCtrl or siGR, or with a GR overexpression plasmid and cultured for 3 d **(B)** Representative confirmation of Affymetrix array results by measurement of significantly altered CXCL8 mRNA expression in primary isolated CAFs using qRT-PCR after single 100 nM Dex, 100 nM Pred, 1 µg/ml Dox, 12 µM RU486, or combination treatment for 1 d. Data represent mean + SEM from 3 independent experiments (one-way ANOVA and correction for multiple testing using Bonferroni's comparison test; **, P< 0.01; ***, P< 0.001). **(C)** Schematic illustration of the treatment protocol for proliferation/viability measurement using epithelial PCa cell lines and collected control and Dex-conditioned PF179T-CAF cell medium. **(D)** Measurement of LNCaP cell proliferation after 4 d treatment with control or Dex conditioned PF179T-CAF cell medium, as well as excluding unspecific Dex mediated effects on cell proliferation, by treating LNCaP cells with increasing concentrations of Dex (0, 1 nM, 10 nM, 100 nM, 1000 nM). Data represent mean + SEM from 3 independent experiments (unpaired t-test; **, P< 0.01; one-way ANOVA and correction for multiple testing using Dunnett's comparison test). **(E)** Presentation of significantly reduced soluble factors using commercially available cytokine and chemokine protein arrays after 3 d 100 nM Dex treatment using primary isolated CAFs from 4 PCa patients. Data represent mean fold change (paired t-test; marked in blue, P< 0.05). **(F)** Altered IL-8 protein expression in primary isolated NAFs after 3 d single/combination treatments with 100 nM Dex, 100 nM Pred, and 6 µM RU486, using specific IL-8-coupled magnetic Luminex ^®^ beads. Data represent mean + SEM from 3 independent experiments (one-way ANOVA and correction for multiple testing using Bonferroni's comparison test; *, P< 0.05; **, P< 0.01; ***, P< 0.001). **(G)** Time course of altered CXCL8 mRNA expression after incubation with 100 nM Dex for 2 h, 4 h, 8 h, and 24 h.

**Figure S3:**

**(A)** Quantification of CXCL8 specific in-situ hybridization microscopy pictures of PF179T-CAF cells after 3 d treatment with 100 nM Dex alone or in combination with 6 µM RU486, as well as microscopy images of prominent stromal CXCL8 mRNA staining within benign and cancerous tissue sections after 3 d 100 nM Dex treatment, using Fiji, an open-source platform for biological-image analysis. **(B)** Measurement of PF179T-CAF cell proliferation/viability after anti-androgen treatment with 5 µM Bic, 5 µM Enza, 5 µM Apa, or 5 µM Daro for 6 d. Data represent mean + SEM from 4 independent experiments (one-way ANOVA and correction for multiple testing using Dunnett's comparison test).

**Figure S4:**

**(A)** CYP17A1 mRNA and protein expression screen using immortalized PF179T-NAF, PF179T-CAF, as well as primary isolated NAFs and CAFs from 3 PCa patients. PF179T-CAF cells transiently transfected with a CYP17A1 overexpression plasmid for 3 d and H295R cells were used as positive controls. **(B)** Altered CXCL8 mRNA expression after single and combination treatment with 100 nM Dex and 2.5 µM Abi for 6 d. Data represent mean + SEM from 5 independent experiments (one-way ANOVA and correction for multiple testing using Bonferroni's comparison test; ***, P< 0.001). **(C)** Schematic illustration of PF179T-CAF cell sub-line generation after single long-term treatment (30 passages; 6 months) with 8 µM Abi, 8 µM Enza, 100 nM Dex and combined long-term 8 µM Abi + 100 nM Dex or 8 µM Enza + 100 nM Dex treatment. **(D)** Representative cell culture microscopy images for altered cell morphology of generated long-term treated PF179T-CAF cell sublines. Magnification: 10x (scale bar = 100 µm). **(E)** GOI mRNA expression of selected up-regulated chemokines (CXCL1, CXCL6) and up-regulated genes connected with cell adhesion (ITGA10, FN1, CLDN7, or IRS2) in generated long-term treated PF179T-CAF cell sub-lines. mRNA data represent mean + SEM from 3 independent experiments (one-way ANOVA and correction for multiple testing using Dunnett's comparison test; *, P<0.05; **, P< 0.01; ***, P< 0.001).

**Figure S5:**

**(A)** Significantly up- and down-regulated genes after 6 d 100 nM Dex or 100 nM Pred treatment, assessed with commercially available PCR gene arrays specifically selected for ECM and adhesion using PF179T-CAF cells. mRNA Data represent mean fold change (paired t-test; marked in red, P< 0.05). **(B)** Confirmation of significantly altered GOI mRNA expression of selected ECM and adhesion markers (CLEC3B, CTGF, VCAN, COL8A1, COL7A1, FN1, ITGA10, ITGA8, ITGA7, CLDN7, MMP1, and MMP3) after 6 d treatment with 100 nM Dex or 100 nM Pred using PF179T-NAF, as well as primary isolated NAFs and CAFs. Data represent mean + SEM from at least 3 independent experiments (one-way ANOVA and correction for multiple testing using Dunnett's comparison test; *, P< 0.05; **, P< 0.01; ***, P< 0.001).

**Figure S6:**

**(A)** Quantification of the adhesion capacity and representative microscopy images of PF179T-CAF cells after short-term culture with 100 nM Pred for 3 d and 6 d and pre-treatment with trypsin for 2 min before measurement. Data represent mean + SEM from 3 independent experiments (one-way ANOVA and correction for multiple testing using Bonferroni's comparison test; *, P< 0.05; **, P< 0.01; ***, P< 0.001). **(B)** Quantification of the adhesion capacity and representative microscopy images of PF179T-NAF cells after short-term culture with 100 nM Dex or 100 nM Pred for 6 d and pre-treatment with trypsin for 2 min before measurement. Data represent mean + SEM from 3 independent experiments (one-way ANOVA and correction for multiple testing using Bonferroni's comparison test; ***, P< 0.001).
